# Ten Hypermethylated lncRNA Genes Are Specifically Involved in the Initiation, Progression, and Lymphatic and Peritoneal Metastasis of Epithelial Ovarian Cancer

**DOI:** 10.3390/ijms252111843

**Published:** 2024-11-04

**Authors:** Eleonora A. Braga, Alexey M. Burdennyy, Leonid A. Uroshlev, Danila M. Zaichenko, Elena A. Filippova, Svetlana S. Lukina, Irina V. Pronina, Iana R. Astafeva, Marina V. Fridman, Tatiana P. Kazubskaya, Vitaly I. Loginov, Alexey A. Dmitriev, Aleksey A. Moskovtsev, Nikolay E. Kushlinskii

**Affiliations:** 1 Institute of General Pathology and Pathophysiology, 125315 Moscow, Russia; burdennyy@gmail.com (A.M.B.); danilamihailovich@mail.ru (D.M.Z.); p.lenyxa@yandex.ru (E.A.F.); sveta_sergeevna349@mail.ru (S.S.L.); zolly_sten@mail.ru (I.V.P.); astafevayr@gmail.com (I.R.A.); loginov7w@gmail.com (V.I.L.); alexey.moscovtsev@gmail.com (A.A.M.); 2 Vavilov Institute of General Genetics, Russian Academy of Sciences, 119991 Moscow, Russia; leoniduroshlev@gmail.com (L.A.U.); marina-free@mail.ru (M.V.F.); 3 N.N. Blokhin National Medical Research Center of Oncology, 115478 Moscow, Russia; oncogen5@ronc.ru (T.P.K.); kne3108@gmail.com (N.E.K.); 4 Engelhardt Institute of Molecular Biology, Russian Academy of Sciences, 119991 Moscow, Russia; alex_245@mail.ru; 5 Russian Medical Academy of Continuing Professional Education, 125993 Moscow, Russia

**Keywords:** epithelial ovarian cancer, lncRNA genes, DNA methylation, primary tumors, peritoneal metastases, EMT-associated genes, miR-137/GAS5-/KCNK15-AS1-/ZNF667-AS1 interactions, Affymetrix HTA 2.0 high-throughput microarrays, overall survival

## Abstract

**Abstract:** Our work aimed to evaluate and differentiate the role of ten lncRNA genes (*GAS5*, *HAND2-AS1*, *KCNK15-AS1*, *MAGI2-AS3*, *MEG3*, *SEMA3B-AS1*, *SNHG6*, *SSTR5-AS1*, *ZEB1-AS1*, and *ZNF667-AS1*) in the development and progression of epithelial ovarian cancer (EOC). A representative set of clinical samples was used: 140 primary tumors from patients without and with metastases and 59 peritoneal metastases. Using MS-qPCR, we demonstrated an increase in methylation levels of all ten lncRNA genes in tumors compared to normal tissues (*p* < 0.001). Using RT-qPCR, we showed downregulation and an inverse relationship between methylation and expression levels for ten lncRNAs (*r_s_* < −0.5). We further identified lncRNA genes that were specifically hypermethylated in tumors from patients with metastases to lymph nodes (*HAND2-AS1*), peritoneum (*KCNK15-AS1*, *MEG3*, and *SEMA3B-AS1*), and greater omentum (*MEG3*, *SEMA3B-AS1*, and *ZNF667-AS1*). The same four lncRNA genes involved in peritoneal spread were associated with clinical stage and tumor extent (*p* < 0.001). Interestingly, we found a reversion from increase to decrease in the hypermethylation level of five metastasis-related lncRNA genes (*MEG3*, *SEMA3B-AS1*, *SSTR5-AS1*, *ZEB1-AS1*, and *ZNF667-AS1*) in 59 peritoneal metastases. This reversion may be associated with partial epithelial–mesenchymal transition (EMT) in metastatic cells, as indicated by a decrease in the level of the EMT marker, CDH1 mRNA (*p* < 0.01). Furthermore, novel mRNA targets and regulated miRNAs were predicted for a number of the studied lncRNAs using the NCBI GEO datasets and analyzed by RT-qPCR and transfection of SKOV3 and OVCAR3 cells. In addition, hypermethylation of *SEMA3B-AS1*, *SSTR5-AS1*, and *ZNF667-AS1* genes was proposed as a marker for overall survival in patients with EOC.

## 1. Introduction

Ovarian cancer (OC) is the most lethal gynecological malignancy and the second most common disease in women after breast cancer [1,2]. As of 2024, there are 314,000 new cases of ovarian cancer diagnosed and 207,000 deaths annually worldwide [2]. OC is a heterogeneous disease, and the most common type is epithelial ovarian cancer (EOC), which also includes five histological subtypes, making diagnosis and treatment challenging [3]. EOC is characterized by an almost asymptomatic course, as a result of which, in most women, it is detected late, at stages with extensive metastasis and chemotherapy resistance, which increases the probability of an unfavorable outcome [4]. Moreover, even when ovarian cancer is detected at an early stage, when there is a response to standard therapy, a relapse develops over time, which quickly develops into a chemoresistant disease [4]. Population screening has proven ineffective, but extensive research in genomics promises new approaches to early diagnosis and prevention of EOC [4].

In addition to lymphogenous and hematogenous metastasis, ovarian carcinogenesis is also characterized by dissemination throughout the peritoneum, metastasis to the greater omentum, and the development of ascites [5]. Moreover, up to 70% of patients with OC have extensive intraperitoneal dissemination at initial diagnosis, with five-year survival rates of less than 20% [1,6]. Metastasis to the abdominal cavity and to the greater omentum is accompanied by the accumulation of ascitic fluid, which is a source of further metastasis [7,8]. The predominance of intraperitoneal carcinomatosis makes the progression of EOC unique, different from the classical and more studied hematogenous and lymphogenous metastasis, which is characteristic of most types of tumors. The mechanism of peritoneal dissemination has been little studied and continues to be studied, including at the morphological, cytological, immunological, and molecular–cellular levels [6,9,10,11].

Recently, the prevailing point of view is that in the processes of cancer progression, the main role is played not so much by genetic disorders but by epigenetic factors, such as DNA and RNA methylation, histone modification, chromatin remodeling, as well as the regulatory effects of non-coding RNAs (ncRNAs) [12,13]. Epigenetic modifications are reversible and can enhance the phenotypic plasticity, which appears to be necessary for the tumor cells, for example, during processes associated with metastasis [13,14,15]. The Human Genome Program and post-genomic studies, which discovered and highlighted the functional role of ncRNAs and their predominance in the transcriptome, allowed us to take a new look at the mechanisms of regulation of biological processes in oncogenesis. The discoveries of miRNAs and then long ncRNAs (lncRNAs) revealed the incredible complexity of transcriptome regulation and raised the understanding of epigenetic mechanisms and regulatory gene networks in malignant tumors to a new level [12,16]. LncRNAs play an important role in regulation of protein-coding gene expression, often interacting with mRNAs through the miRNA-mediated competing endogenous RNA (ceRNA model) mechanism, as both mRNAs and lncRNAs can share binding sites for common miRNAs called miRNA response elements (MREs) [12,16]. The involvement of lncRNAs in miRNA-mediated regulation of protein-coding gene expression has been widely documented in tumors, including OC [17].

Of particular interest is the interaction between two different epigenetic mechanisms in cancer—cross-talk between the regulatory functions of lncRNAs and methylation of genes encoding regulatory lncRNAs, which was observed in cancer [16,18]. In this work, we examined the role of such intersecting epigenetic mechanisms in the pathogenesis of EOC, namely the interaction of lncRNAs with mRNAs and miRNAs against the background of decreased lncRNA expression due to hypermethylation of their genes.

This work is aimed at analyzing changes in methylation of lncRNA genes at different stages of development and progression of EOC, from the onset of cancer to advanced stages with lymphatic and peritoneal metastases, with the development of ascites, and up to consideration of these changes in macroscopic metastases in the peritoneum. Representative clinical material was used, including 140 epithelial ovarian tumors, differentiated by the presence of various types of metastases in patients, as well as 59 macroscopic metastases in the peritoneum.

This work included ten lncRNA genes: *GAS5*, *HAND2-AS1*, *KCNK15-AS1*, *MAGI2-AS3*, *MEG3*, *SEMA3B-AS1*, *SNHG6*, *SSTR5-AS1*, *ZEB1-AS1*, and *ZNF667-AS1*. For the *SNHG6* gene (Small nucleolar RNA host gene 6) and *GAS5* gene (Growth arrest specific 5/Small nucleolar RNA host gene 2), we previously obtained preliminary data on their hypermethylation in EOC [19,20]. Eight other genes were bioinformatically selected as hypermethylated using the NCBI Gene Expression Omnibus (GEO) datasets GSE81228 and GSE146556, containing whole-genome bisulfite sequencing data for OC samples. MEG3 (Maternal gene expression 3) was an intergenic lncRNA. Seven other lncRNAs were antisense transcripts, namely HAND2-AS1 (Heart and neural crest derived expressed 2 antisense RNA 1), KCNK15-AS1 (Two-pore potassium channel domain subfamily member 15 antisense RNA 1; also known as WNT1-inducible signaling pathway protein 2 (WISP2) antisense RNA 1; or RP11-445H22.4 in larger datasets), MAGI2-AS3 (MAGI2 antisense RNA 3), SEMA3B-AS1 (Semaphorin-3B antisense RNA 1), SSTR5-AS1 (Somatostatin receptor subtype 5 antisense RNA 1), ZEB1-AS1 (Zinc finger E-box binding homeobox 1 antisense RNA 1), and ZNF667-AS1 (Zinc finger protein 667 antisense RNA 1, also known as Mortal obligate RNA transcript, MORT). Of the lncRNA genes selected for this study, DNA methylation in EOC has previously been shown for *MEG3* [21], *GAS5*, *SEMA3B-AS1*, *SNHG6*, and *ZNF667-AS1* in our preliminary studies [19,20]. Epigenetic inactivation of *HAND2-AS1* has also been demonstrated by Gokulnath et al. [22]. There was no information about hypermethylation in EOC for *KCNK15-AS1*, *MAGI2-AS3*, *SSTR5-AS1*, and *ZEB1-AS1* (NCBI PubMed, https://pubmed.ncbi.nlm.nih.gov/, accessed on 1 October 2024).

In this work, we investigated methylation and expression changes of ten lncRNA genes in a representative set of EOC samples and assessed the effect of methylation on expression downregulation in tumors, as well as the association of methylation status with clinical and morphological parameters and the presence of different types of metastases. The analysis was also performed in 59 macroscopic peritoneal metastases, considering the primary importance of intraperitoneal carcinomatosis in ovarian malignancies. In addition, we evaluated the prognostic value of lncRNA gene hypermethylation as a marker of metastasis and decreased survival. We also searched for probable mRNAs and miRNAs interacting with these ten lncRNAs using predictive and experimental approaches.

## 2. Results

### 2.1. Hypermethylated lncRNA Genes Are Involved in EOC Occurrence

**Figure 1:**
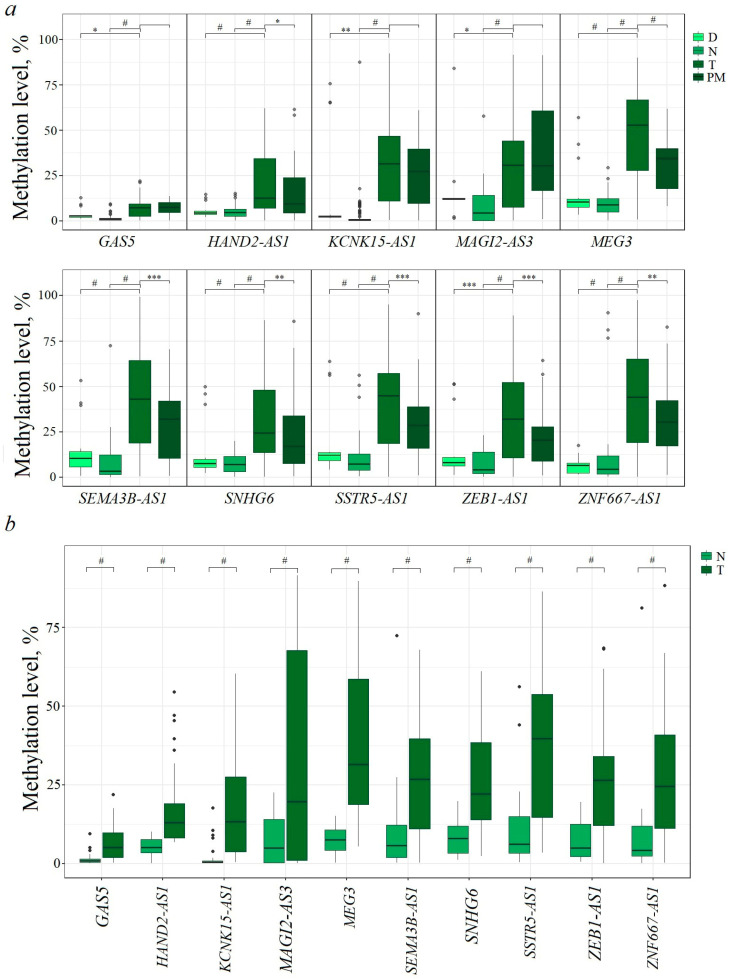
(**a**) Methylation levels of ten lncRNA genes in 18 samples from donors (D), 123 histologically normal ovarian tissues from EOC patients (N), 140 primary ovarian tumors (T), and 59 peritoneal macroscopic metastases (PM); (**b**) methylation levels of ten lncRNA genes in 43 primary ovarian tumors from patients without metastases (T) and 43 matched histologically normal ovarian tissues (N). * *p* < 0.05, ** *p* < 0.01, *** *p* < 0.001, # *p* < 0.0001.

Methylation levels of ten lncRNA genes (*GAS5*, *HAND2-AS1*, *KCNK15-AS1*, *MAGI2-AS3*, *MEG3*, *SEMA3B-AS1*, *SNHG6*, *SSTR5-AS1*, *ZEB1-AS1*, and *ZNF667-AS1*) were determined in 140 EOC samples with and without metastases (T), 123 matched histologically normal tissue samples (N), 59 peritoneal metastases (PM), and 18 ovarian tissue samples from post-mortem “donors” with no history of cancer (D) (Figure 1a).

Statistically significant increases in methylation levels were found in tumor samples compared to matched normal ovarian tissues and deceased donor ovarian tissues for all ten lncRNA genes (*p* < 0.001, Figure 1a). It is important to highlight that DNA hypermethylation of lncRNA genes *HAND2-AS1*, *KCNK15-AS1*, *SSTR5-AS1*, and *ZEB1-AS1* in EOC is reported here for the first time.

In addition, there was a statistically significant (*p* ≤ 0.001) reduced level of methylation of five lncRNA genes (*MEG3*, *SEMA3B-AS1*, *SSTR5-AS1*, *ZEB1-AS1*, and *ZNF667-AS1*) in 59 peritoneal metastases compared to their methylation level in 140 primary tumors (Figure 1a) that indicated the complex nature of the relationship between the metastasis process and methylation of these genes.

To assess the role of methylation of these genes specifically in the occurrence of EOC and exclude the influence of progression and metastasis, analysis of methylation levels was carried out in a set of 43 paired (tumor/normal, T/N) samples from patients without metastases (Figure 1b). A statistically significant increase in methylation in tumors of patients without metastases was detected for all ten examined lncRNA genes (*p* < 0.001, Figure 1b), indicating a connection between the methylation of these genes and the pre-metastatic stage. It was of interest to evaluate the involvement of methylation of these ten genes in the processes of progression and metastasis of EOC.

### 2.2. Relationship of Methylation of Examined lncRNA Genes with Clinical Stage and Histological Grade of EOC

**Figure 2:**
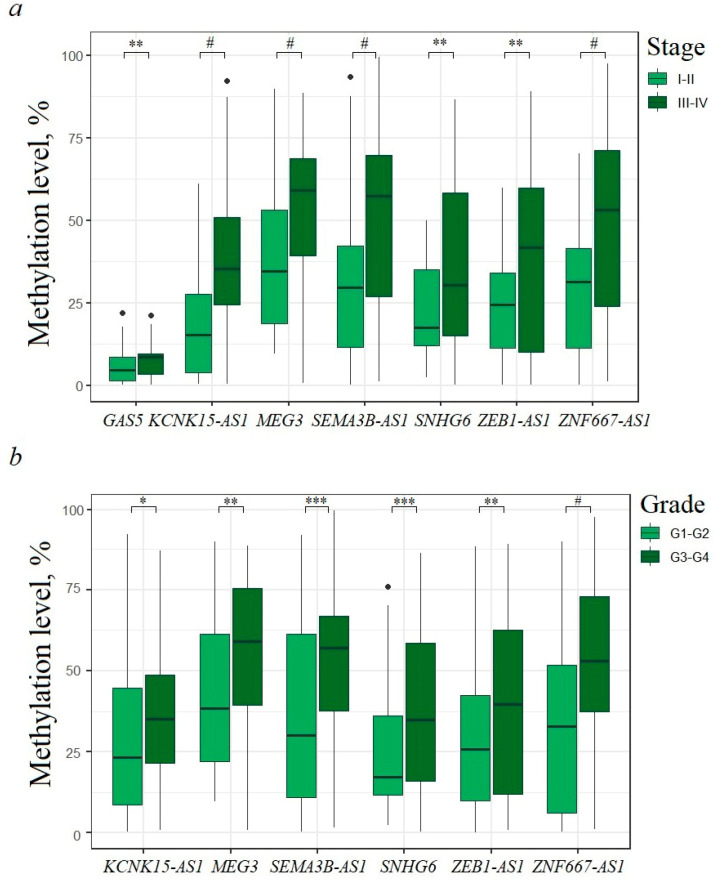
(**a**) Hypermethylated lncRNA genes associated with advanced clinical stages of EOC; 47 samples of stages I + II and 93 samples of stages III + IV; (**b**) hypermethylated lncRNA genes associated with advanced histological grade of EOC; 72 G1–G2 samples and 68 G3–G4 samples. * *p* < 0.05, ** *p* < 0.01, *** *p* < 0.001, # *p* < 0.0001.

Comparison of methylation levels at early (I + II) and more advanced (III + IV) clinical stages of EOC showed a statistically significant (*p* < 0.01) increase in the methylation level of seven lncRNA genes (*GAS5*, *KCNK15-AS1*, *MEG3*, *SEMA3B-AS1*, *SNHG6*, *ZEB1-AS1*, and *ZNF667-AS1*) at late stages (Figure 2a). The most statistically significant (*p* < 0.0001) relationship was found for four genes: *KCNK15-AS1*, *MEG3*, *SEMA3B-AS1*, and *ZNF667-AS1* (Figure 2a).

Comparison of 51 T1–T2 samples with 89 T3 samples revealed a pattern identical to that in the analysis of dependence on stage: a statistically significant (*p* < 0.01) increase in methylation of the same seven genes, with the most statistically significant increase (*p* < 0.0001) for the same four genes: *KCNK15-AS1*, *MEG3*, *SEMA3B-AS1*, and *ZNF667-AS1*.

We further compared 72 samples of primary ovarian tumors with histological grade G1–G2 and 68 samples with histological grade G3–G4. Six genes (*KCNK15-AS1*, *MEG3*, *SEMA3B-AS1*, *SNHG6*, *ZEB1-AS1*, and *ZNF667-AS1*) were found to be associated (*p* < 0.05) with the histological grade of EOC based on their methylation levels (Figure 2b). Of these, three genes (*SEMA3B-AS1*, *SNHG6*, and *ZNF667-AS1*) were most statistically significantly (*p* < 0.001) involved in increasing the degree of malignancy, especially *ZNF667-AS1* (*p* < 0.0001).

It can be noted that seven hypermethylated lncRNA genes were associated at *p* < 0.01 with advanced stages, and only five genes (*MEG3*, *SEMA3B-AS1*, *SNHG6*, *ZEB1-AS1*, and *ZNF667-AS1*) were associated with histological grade to the same extent (*p* < 0.01). Two lncRNA genes out of ten examined, *SEMA3B-AS1* and *ZNF667-AS1*, were most (*p* < 0.0001) involved in EOC progression, including clinical stage, tumor size, and histological grade.

### 2.3. Different lncRNA Gene Sets Are Specifically Involved in Different Types of Metastases, Such as Lymph Node, Peritoneum, and Greater Omentum

**Figure 3:**
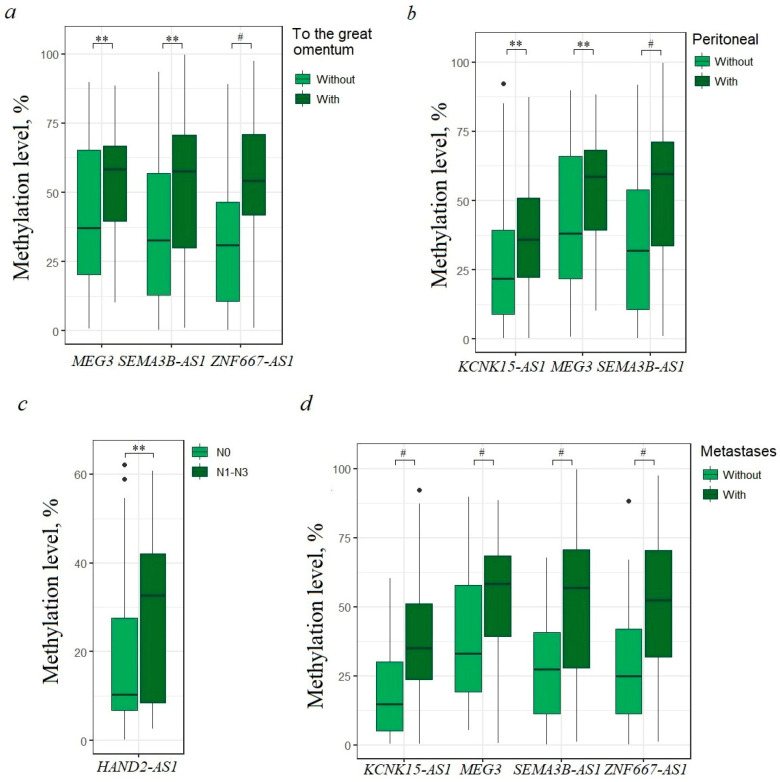
Hypermethylated lncRNA genes associated with different types of EOC metastases: (**a**) metastases to the great omentum (70 patients—without, 70 patients—with); (**b**) dissemination through the peritoneum (70 patients—without, 70 patients—with); (**c**) metastases to the lymph nodes (110 patients—N0, 30 patients—N1–N3); (**d**) samples from patients with metastases of any type were considered (44 patients—without, 96 patients—with). ** *p* < 0.01, # *p* < 0.0001.

Next, it was of interest to study whether there were specific differences in methylation levels of any of the lncRNA genes under study in tumors of patients with and without metastases. Because EOC is characterized by different types of metastases, such as distant organs, lymph nodes, peritoneum, and great omentum, we studied all these types of EOC dissemination. It turned out that in tumors of patients with metastases to the great omentum, a statistically significant (*p* < 0.01) increase in the methylation level was observed for three lncRNA genes: *MEG3*, *SEMA3B-AS1*, and *ZNF667-AS1*, and highly statistically significant (*p* < 0.0001) hypermethylation was shown for *ZNF667-AS1* (Figure 3a).

Another set of three genes (*KCNK15-AS1*, *MEG3*, *SEMA3B-AS1*) statistically significantly (*p* < 0.01) increased methylation in tumors of patients with dissemination into the abdominal cavity, and the methylation of *SEMA3B-AS1* was highly statistically significantly (*p* < 0.0001) associated with peritoneal dissemination (Figure 3b). The methylation level of *HAND2-AS1* was associated (*p* < 0.01) with lymphogenous metastasis (Figure 3c). Thus, each of the three types of metastases was characterized by its own set of markers and by individual specific markers: metastases to the lymph nodes—*HAND2-AS1*, to the peritoneum—*SEMA3B-AS1*, and to the omentum—*ZNF667-AS1*.

Finally, when considering all types of EOC metastasis, including lymph node metastasis, distant metastasis, peritoneal metastasis, and omental metastasis, four lncRNA genes (*KCNK15-AS1*, *MEG3*, *SEMA3B-AS1*, and *ZNF667-AS1*) were highly associated (*p* < 0.0001), and thus these four genes may represent a significant set of markers for the prognosis of any EOC metastasis (Figure 3d).

The spread of metastases to the peritoneum and omentum is associated with the accumulation of ascitic fluid. According to the data obtained, in tumors of 61 patients with ascites, an increase in hypermethylation of the *MEG3* and *SEMA3B-AS1* genes was noted (*p* < 0.05), although the association was less significant than with metastases.

It is interesting to note that both at advanced stages and in metastasis, the same four genes most reliably (*p* < 0.0001) increased methylation, *KCNK15-AS1*, *MEG3*, *SEMA3B-AS1*, and *ZNF667-AS1*, and are obviously factors in the progression of EOC.

### 2.4. Hypermethylated lncRNA Genes Specifically Involved in the Formation of Macroscopic Metastases in the Peritoneum of EOC Patients

**Figure 4:**
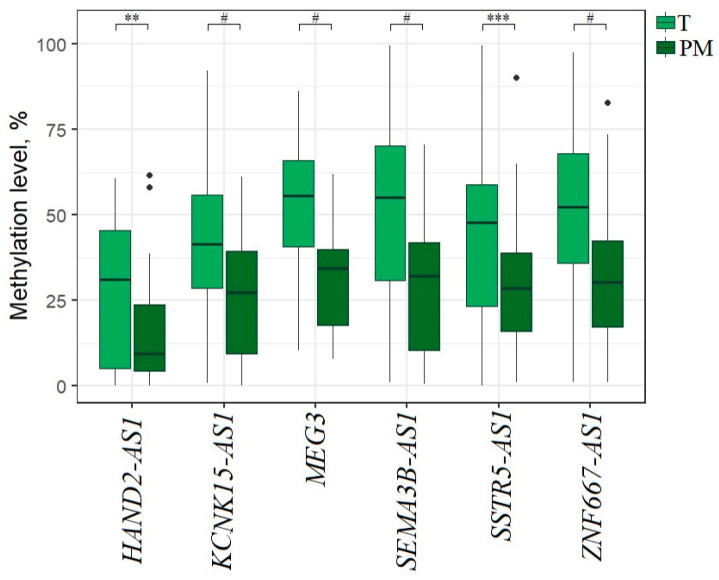
Methylation levels of six lncRNA genes in 59 peritoneal metastases (PM) vs. 59 primary tumors from the same EOC patients. ** *p* < 0.01, *** *p* < 0.001, # *p* < 0.0001.

Next, we paid attention to the influence of hypermethylated lncRNA genes on the transition from primary tumors of patients with metastases to formed macroscopic metastases colonized in the peritoneum. In a separate figure, we present data for 59 such metastases compared to 59 primary tumors from the same patients; this pattern shows a statistically significant (*p* < 0.01) decrease in methylation level of six lncRNA genes (Figure 4).

It is important to emphasize that five genes (*HAND2-AS1*, *KCNK15-AS1*, *MEG3*, *SEMA3B-AS1*, and *ZNF667-AS1*) showed both a statistically significant increase in methylation levels in primary tumors with different types of metastases and a statistically significant decrease in methylation levels in macroscopic metastases instead of the expected further increase (compare Figure 3 and Figure 4).

To explain this phenomenon, it was necessary to answer two questions: (a) were the changes in the methylation level of these lncRNA genes accompanied by changes in expression; (b) how have the properties of tumor cells changed during the transition from primary tumors to macroscopic peritoneal metastases? We have tried to answer these questions in the following sections.

### 2.5. Functional Significance of Methylation of Ten lncRNA Genes in the Regulation of Their Expression in EOC

**Figure 5:**
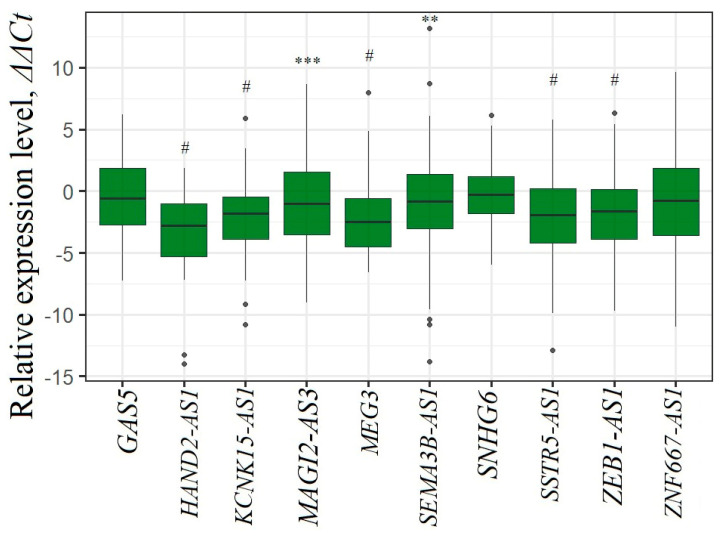
Changes in the levels of ten lncRNAs in primary tumors compared to matched histologically normal tissues. The lncRNAs HAND2-AS1, MEG3, and ZEB1-AS1 were tested in the subset of 73 paired (T/N) samples, GAS5 in 68 samples, and the remaining six lncRNAs in 56 samples. ** *p* < 0.01, *** *p* < 0.001, # *p* < 0.0001.

The functional role of aberrant methylation of lncRNA genes was tested by the effect of methylation on the expression level of ten lncRNAs (GAS5, HAND2-AS1, KCNK15-AS1, MAGI2-AS3, MEG3, SEMA3B-AS1, SNHG6, SSTR5-AS1, ZEB1-AS1, and ZNF667-AS1) in a subset of paired (T/N) EOC samples. The data on the change in levels of ten lncRNAs in ovarian tumor samples relative to normal tissues are shown in Figure 5.

As shown in Figure 5, all ten lncRNAs showed downregulation, of which five lncRNAs (HAND2-AS1, KCNK15-AS1, MEG3, SSTR5-AS1, and ZEB1-AS1) showed the most statistically significant downregulation (*p* < 0.0001). MAGI2-AS3 showed downregulation with *p* < 0.001 and SEMA3B-AS1 showed downregulation with *p* < 0.01.

These data were in good agreement with the data on hypermethylation of genes encoding these lncRNAs and demonstrated the functional significance of methylation in downregulating the expression of these lncRNAs in EOC.

**Figure 6:**
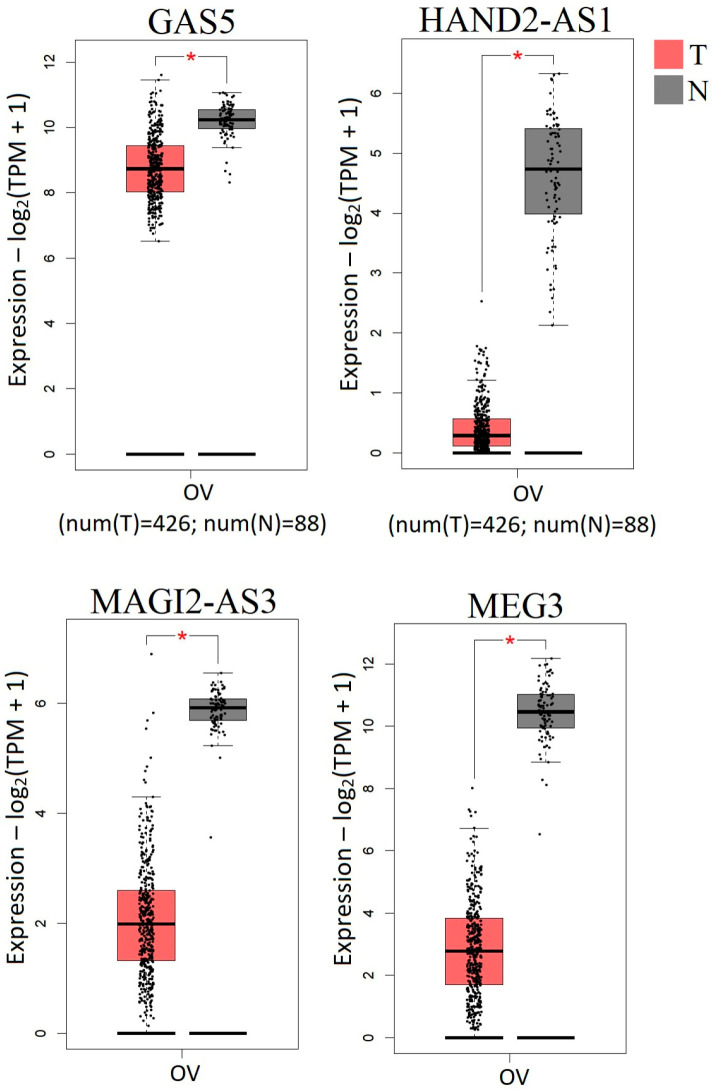
Changes in expression levels of four lncRNAs (GAS5, HAND2-AS1, MAGI2-AS3, MEG3) in serous ovarian cystadenocarcinoma according to the GEPIA 2.0 data (red—tumor, gray—normal); 426 tumor samples, 88 normal tissues; red asterisk corresponds to *p* < 0.01; TPM—transcripts per million.

Our data were also consistent with the expression data of these lncRNAs from the GEPIA 2.0 (Gene Expression Profiling Interactive Analysis, http://gepia2.cancer-pku.cn/#index, accessed on 1 August 2024) database (Figure 6).

According to GEPIA 2.0, four lncRNAs—GAS5, HAND2-AS1, MAGI2-AS3, and MEG3—showed statistically significant downregulation (*p* < 0.01, Figure 6). In addition, we found highly statistically significant downregulation of three more lncRNAs: KCNK15-AS1, SSTR5-AS1, and ZEB1-AS1 in EOC (*p* < 0.0001, Figure 5).

**Figure 7:**
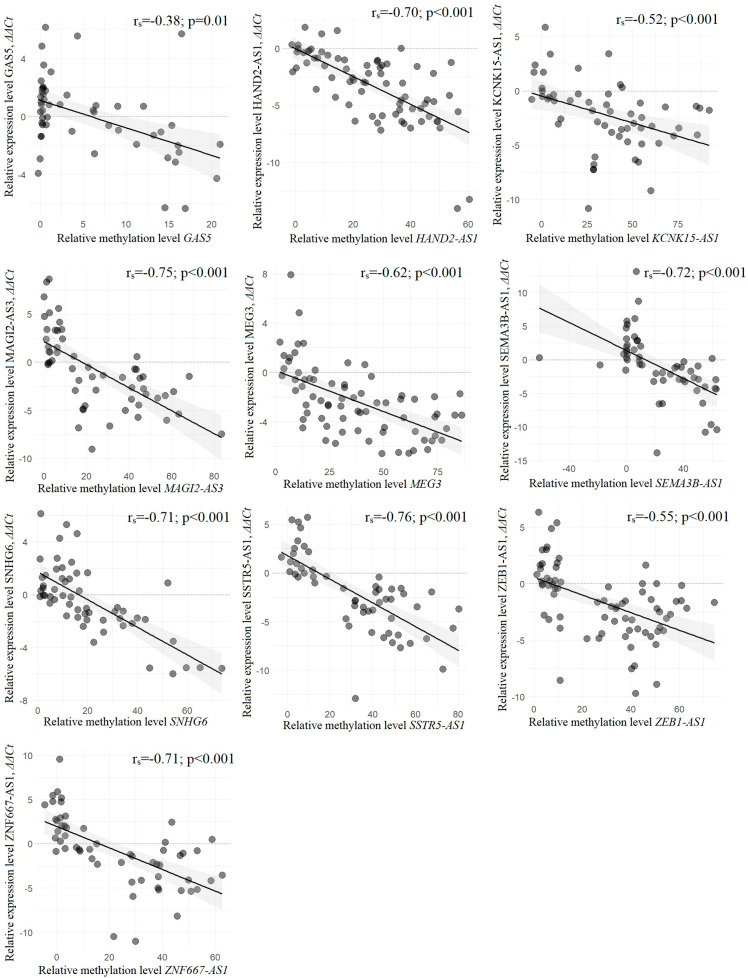
Statistically significant negative correlation between the changes in methylation and expression levels of ten lncRNA genes in the subset of 90 paired (T/N) samples of EOC. The lncRNAs HAND2-AS1, MEG3, and ZEB1-AS1 were tested in the subset of 73 samples, GAS5 in 68 samples, and the other six lncRNAs in 56 samples. Spearman’s correlation coefficients (*r_s_*) are given.

Next, the relationship between methylation and expression of ten lncRNAs was characterized using Spearman’s correlation coefficient and correlation plots (Figure 7).

Statistically significant negative correlations between methylation level and expression level were shown for all ten lncRNAs, but the most significant (*r_s_* from −0.52 to −0.76, *p* < 0.001) was found for nine lncRNAs, except GAS5, for which a slightly lower but also statistically significant negative correlation (*r_s_* = −0.38, *p* = 0.01) was found. This result indicated the functional significance of hypermethylation in downregulation of all ten lncRNA genes examined in EOC.

### 2.6. Metastasis-Associated LncRNAs May Be Involved in Partial EMT

**Figure 8:**
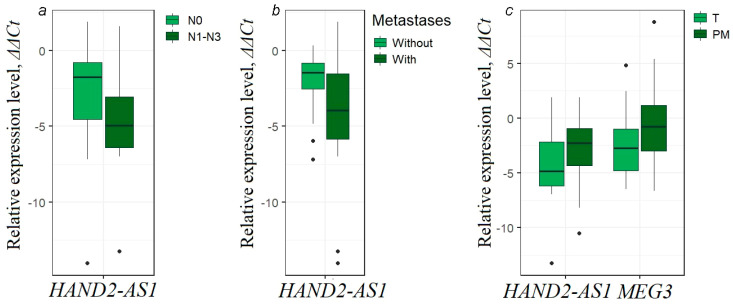
(**a**) Decreased relative expression level of lncRNA HAND2-AS1 in primary ovarian tumors from patients with lymphatic metastases (21 samples, T/N) compared to primary tumors without lymphatic metastases (52 samples, T/N); (**b**) decreased relative expression level of lncRNA HAND2-AS1 in primary ovarian tumors from patients with any metastases (50 samples, T/N) compared to primary tumors without any metastases (23 samples, T/N); (**c**) increased relative expression level of lncRNAs HAND2-AS1 and MEG3 in peritoneal metastases compared to primary tumors from the same EOC patients (31 PM samples vs. 31 tumor samples).

As shown above, five lncRNA genes out of ten studied (*HAND2-AS1*, *KCNK15-AS1*, *MEG3*, *SEMA3B-AS1*, and *ZNF667-AS1*) were mostly associated with EOC metastasis, and moreover, these genes statistically significantly (*p* < 0.01) increased methylation levels in primary tumors with different types of metastases (Figure 3), but in macroscopic metastases, they statistically significantly (*p* < 0.01) decreased methylation levels (Figure 4). Since a clear inverse relationship was established between the levels of methylation and expression for these lncRNAs, we next investigated the possible association between different types of metastases and the expression levels of these five lncRNAs. We obtained data on the association with metastasis for lncRNAs HAND2-AS1 and MEG3, the expression level of which was determined in the subset of 73 paired (T/N) EOC samples (Figure 8).

A highly statistically significant decrease (*p* = 0.001) in the expression level of HAND2-AS1 was shown in primary tumors of patients with lymph node metastases compared to non-metastatic tumors (Figure 8a). A statistically significant decrease (*p* = 0.013) in the expression level of lncRNA HAND2-AS1 was also found in primary tumors with total metastasis, taking into account any types of metastases (Figure 8b). These data further confirmed the association of *HAND2-AS1* gene hypermethylation with its downregulation in EOC. Moreover, using the example of *HAND2-AS1*, it was shown that both an increase in lncRNA gene hypermethylation in primary tumors and a decrease in its expression were associated with the metastasis process.

Furthermore, when comparing the expression in 31 peritoneal metastases with that in 31 primary tumors from the same EOC patients, we observed an obvious increase in the expression levels of lncRNAs HAND2-AS1 and MEG3 in peritoneal metastases (*p* < 0.05, Figure 8c). Thus, comparing the pattern in PM with primary tumors during metastasis, instead of a further decrease in expression in PM, there was a statistically significant increase in HAND2-AS1 and MEG3 expression (Figure 8c). This is similar to the reversion from the increase in methylation levels of five lncRNA genes (*HAND2-AS1*, *KCNK15-AS1*, *MEG3*, *SEMA3B-AS1*, and *ZNF667-AS1*) in primary tumors during metastasis to the decrease in their methylation levels in PM (Figure 4).

**Figure 9:**
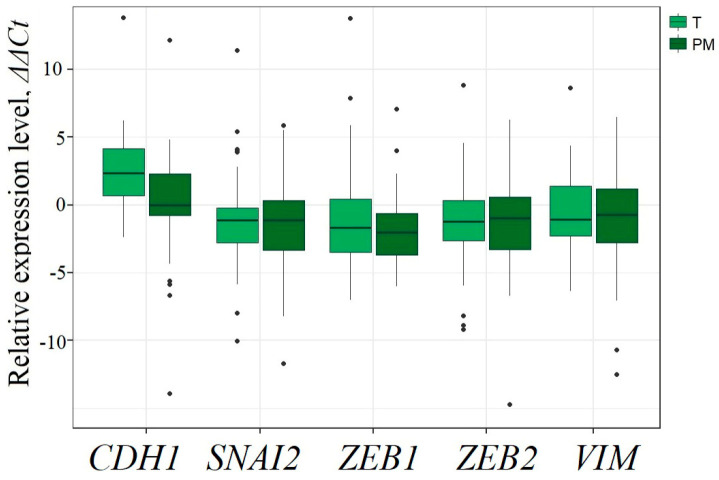
Changes in mRNA levels of five EMT markers (CDH1, SNAI2/SLUG, ZEB1, ZEB2, VIM mRNAs) in 30 peritoneal metastases (PM) compared to 46 primary ovarian tumors (T).

It is known that during the formation of micro- or macroscopic metastases, an increase in mesenchymal characteristics may occur to increase the mobility of tumor cells. It can be assumed that these changes in the state of cells are associated with changes in the levels of expression and/or methylation of some lncRNAs. To assess the change in epithelial and mesenchymal characteristics during the transition from primary tumors to peritoneal metastases, epithelial–mesenchymal transition (EMT) markers (E-cadherin and vimentin mRNAs) and EMT transcription factors (ZEB1, ZEB2, SNAI2/SLUG mRNAs) were tested (Figure 9).

A statistically significant decrease in the level of CDH1 (*p* = 0.005) was established in peritoneal metastases (Figure 9) that suggested partial EMT during the transition from primary tumor cells to metastatic cells. Based on all these data, it was possible to suggest the participation of five lncRNAs (HAND2-AS1, KCNK15-AS1, MEG3, SEMA3B-AS1, and ZNF667-AS1) in the process of peritoneal metastasis formation via partial EMT transition. In this regard, a predictive assessment of the presence of genes associated with EMT was carried out among the genes in the regulation of which five metastasis-associated lncRNAs may participate.

### 2.7. Identification of Potential Target mRNAs for the Studied lncRNAs and Their Association with EMT Genes; Identification of Potentially Regulated miRNAs

Evaluation of potential target mRNAs for the studied ten lncRNAs was performed using the dataset from NCBI GEO (GSE211669) for 131 patients of serous ovarian carcinoma and Spearman’s correlation analysis (Supplementary Tables S1–S11). These mRNAs may represent direct or, more often, indirect targets, although they may also perform regulatory functions themselves. For the majority of lncRNAs, mainly positive correlation was observed, and for SEMA3B-AS1, negatively correlated mRNAs were also widely represented and taken into account. The data from the GeneCards and dbEMT 2.0 databases were used to select EMT-associated genes. Results for a number of lncRNAs are given in Table 1.

We saw that of the positively correlated mRNAs, 15 to 42% were associated with EMT according to GeneCards, and 2 to 6% according to dbEMT 2.0. We found that lncRNAs HAND2-AS1, KCNK15-AS1, MAGI2-AS3, MEG3, SEMA3B-AS1, and ZNF667-AS1 were highly associated with EMT genes and shared common EMT-related target genes. The target gene lists of HAND2-AS1, MAGI2-AS3, and MEG3 by GeneCards included typical EMT drivers, such as ZEB1, ZEB2, SNAI2, etc.

Thus, the observed decrease in the methylation level of a number of lncRNA genes in peritoneal metastases compared to primary tumors of the same EOC patients (Figure 4), as well as an increase in the expression level of HAND2-AS1 and MEG3 in peritoneal metastases (Figure 8c) may indeed correspond to partial EMT.

Screening of mRNAs interacting with lncRNA based on correlation analysis using the GSE211669 data (Supplementary Tables S1–S11) was then continued by local sequence alignment of mRNA and lncRNA sequences using the NCBI Nucleotide Archive and the Smith–Waterman algorithm. The top ten results are shown in Table 2; all results are shown in Supplementary Table S21.

**Figure 10:**
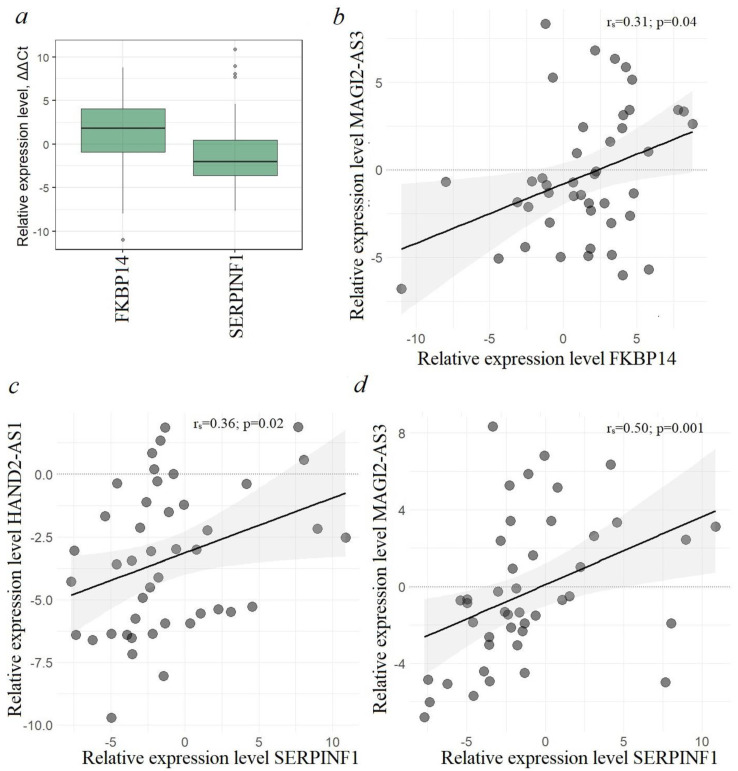
(**a**) Relative expression levels of FKBP14 and SERPINF1 mRNAs in the subset of 44 EOC samples (27 T/N +17 PM/N); (**b**–**d**) positive correlations of expression levels of lncRNAs MAGI2-AS3 and HAND2-AS1 with expression levels of FKBP14 and SERPINF1 mRNAs in 44 EOC samples (27 T/N +17 PM/N).

For the experimental analysis, we selected the *SERPINF1* gene, which showed a high positive correlation and high complementarity with the two studied lncRNAs—HAND2-AS1 and MAGI2-AS3—as well as the *FKBP14* gene, which also showed a high correlation and complementarity with MAGI2-AS3 and a high correlation, although significantly less complementarity, with HAND2-AS1 (Table 2). The relative expression levels of FKBP14 and SERPINF1 mRNAs and the results of the analysis of their possible correlations with lncRNAs HAND2-AS1 and MAGI2-AS3 are shown in Figure 10.

In ovarian tumor samples, we observed decreased expression of SERPINF1 and increased expression of FKBP14, which is consistent with the data on the downregulation and antiangiogenic and tumor suppressive activity of SERPINF1 in cervical cancer [23] and, conversely, on the upregulation and oncogenic properties of FKBP14 in OC [24]. RT-qPCR and Spearman’s correlation methods (Figure 10b–d) confirmed the possible interaction of SERPINF1 mRNA with lncRNAs HAND2-AS1 and MAGI2-AS3, as well as FKBP14 mRNA with MAGI2-AS3, which was in accordance with the bioinformatics data (Table 2). This result, however, requires confirmation by direct binding methods such as luciferase assay, etc. Nevertheless, it can be concluded from these observations that out of thousands of mRNAs positively correlated with lncRNA, we see only a few examples of mRNAs that are putatively involved in direct interactions with lncRNA. An alternative and most frequently experimentally confirmed mechanism of lncRNA influence on protein-coding gene expression is mediated by miRNA according to the competing endogenous RNA (ceRNA) model: lncRNA/miRNA/mRNA, including in OC [25].

MiRNAs negatively correlated with ten studied lncRNAs were screened using the GSE119055 dataset (https://www.ncbi.nlm.nih.gov/geo/query/acc.cgi?acc=GSE119055, accessed on 1 August 2024) for six patients of serous ovarian carcinoma and Spearman’s correlation method was used (*r_s_* < −0.6). The results (2801 lncRNA/miRNA interactions) are given in Supplementary Table S22. Four miRNAs (miR-124-3p, miR-124-5p, miR-137-3p, and miR-33b-5p) that showed negative correlations with all ten lncRNAs were selected for further analysis (Table 3). Pairs with complementarity sites were determined by local sequence alignment of miRNA and lncRNA sequences (Table 3 and Supplementary Table S23).

**Figure 11:**
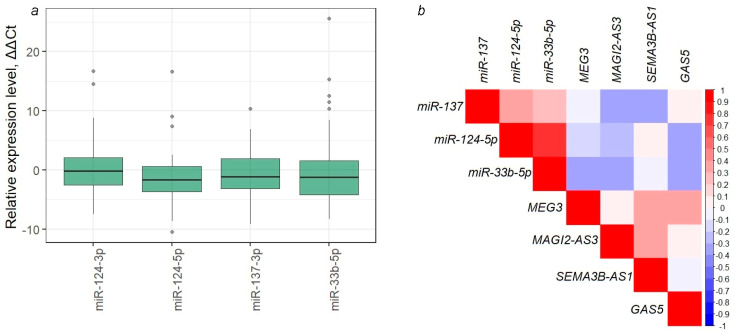
(**a**) Relative expression levels of four miRNAs (miR-124-3p, miR-124-5p, miR-137-3p, miR-33b-5p); (**b**) correlation plot to analyze possible correlations between four miRNAs and ten lncRNAs in the subset of 41 paired (T/N) EOC samples.

To experimentally test possible interactions between the studied lncRNAs and four predicted miRNAs (mir-124-3p, mir-124-5p, mir-137-3p, and mir-33b-5p), the expression profiles of these miRNAs were determined in the subset of 41 paired (T/N) EOC samples, and possible correlations with lncRNAs were analyzed (Figure 11).

A statistically significant decrease in the expression level of miR-124-5p (*p* = 0.0004), miR-137-3p (*p* = 0.005), and miR-33b-5p (*p* = 0.004) in ovarian tumors was shown (Figure 11a), which may be also due to the hypermethylation of miRNA genes themselves [26,27]. Five statistically significant negative correlations between miRNAs and lncRNAs were determined for pairwise combinations: GAS5/miR-124-5p, MAGI2-AS3/miR-137-3p, MAGI2-AS3/miR-33b-5p, MEG3/miR-33b-5p, SEMA3B-AS1/miR-137-3p (*r_s_* < −0.3, *p* < 0.05), as well as at the trend level for the GAS5/miR-33b-5p (*r_s_* = −0.3, *p* = 0.06) (Figure 11b). Moreover, four statistically significant correlations corresponded to those predicted bioinformatically: GAS5/miR-124-5p, MAGI2-AS3/miR-33b-5p, MEG3/miR-33b-5p, SEMA3B-AS1/miR-137-3p (Table 3). For the pair MAGI2-AS3/miR-33b-5p, the presence of a complementarity site, 7mer-m8, was also predicted.

In addition, a pilot comparison of the relative expression levels of four miRNAs and three mRNAs (VIM, ZEB1, SNAI2/SLUG) (Figure 9) in a total set of 46 EOC samples also revealed negatively correlated pairs: miR-124-3p/SNAI2, miR-124-3p/VIM, miR-124-3p/ZEB1 (*r_s_* ≤ −0.4, *p* < 0.01) and miR-33b-5p/VIM, miR-33b-5p/ZEB1 (*r_s_* ≤ −0.4, *p* < 0.05), which allowed us to construct a hypothetical triplet MAGI2-AS3/miR-33b-5p/VIM, ZEB1. Moreover, positive correlations of MAGI2-AS3 with VIM and ZEB1 were predicted bioinformatically (*r_s_* > 0.6, *p* < 10^−13^, Supplementary Table S9), and the correlation of MAGI2-AS3 with ZEB1 was also detected by RT-qPCR (*r_s_* > 0.3, *p* < 0.05).

Our putative MAGI2-AS3/miR-33b-5p/ZEB1 triplet was not described, while the activating effect of MAGI2-AS3 on ZEB1 via miR-141/200a has been previously shown in gastric cancer [28].

### 2.8. Identification of Potential lncRNAs/miRNAs Interactions Using OVCAR3 and SKOV3 Cell Culture Transfection in Combination with Affymetrix and RT-qPCR

We transfected SKOV3 and OVCAR3 cells with miR-124-3p and miR-137-3p mimics and analyzed differentially expressed lncRNAs and mRNAs using Affymetrix HTA 2.0 high-throughput microarrays. The levels of mRNA downregulation in response to transfection of SKOV3 cells with the miR-137-3p and miR-124-3p mimics were comparable (1.05–1.6-fold, *p* < 0.3), although miR-124-3p downregulated a larger number of mRNAs than miR-137-3p: 94 and 42 mRNAs, respectively (Table 4, Table 5 and Table 6). It is noteworthy that the lncRNA responses to transfection of OVCAR3 with miR-137-3p mimic (28 lncRNAs, 1.05–1.3-fold, *p* < 0.3) and SKOV3 cells with the miR-124-3p mimic (30 lncRNAs, 1.05–1.6-fold, *p* < 0.3) were comparable.

Among the ten hypermethylated lncRNAs examined in this work, a transfection response was detected for GAS5; moreover, a GAS5 response was obtained to transfection of miRNA mimics of both miR-124-3p and miR-137-3p (Table 4, Table 5 and Table 6). The highest GAS5 response was found in SKOV3 cells to the effect of miR-124-3p (1.27-fold, *p* < 0.01).

From the predicted lncRNA/miRNA interactions (Table 3), we selected the most abundant miRNAs (miR-137-3p and miR-124-3p) with a large number of mapped reads in the miRbase data for further studies. Transfections of SKOV3 and OVCAR3 cells with the miR-137-3p and miR-124-3p mimics in combination with RT-qPCR were used to test interactions of these two miRNAs with any of the ten lncRNAs in OC.

**Figure 12:**
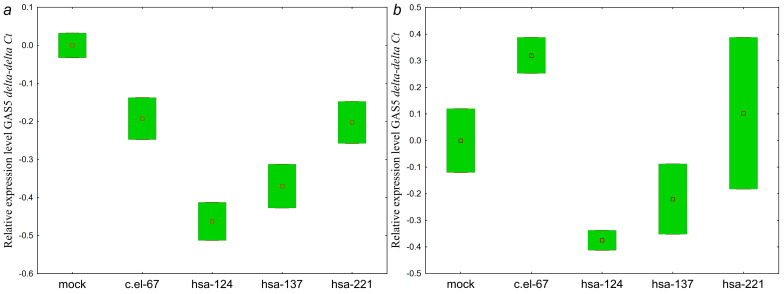
Changes in the levels of lncRNA GAS5 in (**a**) SKOV3 and (**b**) OVCAR3 cells transfected with miRNA mimics: c.el-67—cel-miR-67-3p, hsa-124—hsa-miR-124-3p, hsa-137—hsa-miR-137-3p. SKOV3: *p* (hsa-124 vs. mock) = 0.13, *p* (hsa-137 vs. mock) = 0.32, *p* (hsa-221 vs. mock) = 0.99; OVCAR3: *p* (hsa-124 vs. mock) = 0.99, *p* (hsa-137 vs. mock) = 0.99, *p* (hsa-221 vs. mock) = 0.99. Data are presented as the median and 25–75% percentiles (n = 4).

As can be seen from Figure 12, hsa-miR-124-3p, hsa-miR-137-3p miRNAs cause a small and similar decrease in the GAS5 level. Although the significance of the observed changes is low, the similar patterns of downregulation in the two lines transfected with hsa-miR-137-3p mimics suggest a link between hsa-miR-137-3p and GAS5, which is consistent with the bioinformatic prediction (Table 3).

GAS5 interactions with miR-124(-3p) and miR-137(-3p) in OC have not been previously reported, although there is evidence of GAS5 involvement in the suppression of melanoma cell proliferation, migration, and invasion via the GAS5/miR-137 axis [29].

We tested the bioinformatically predicted association between lncRNA ZNF667-AS1 and miR-137-3p.

**Figure 13:**
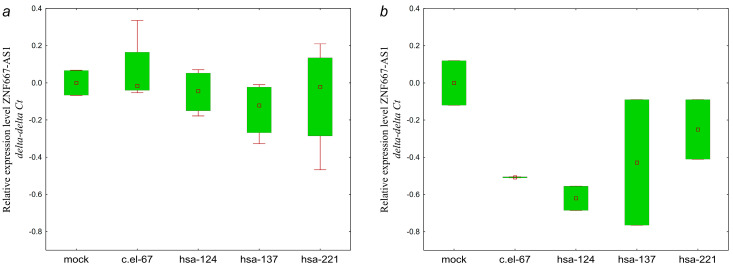
Changes in the levels of lncRNA ZNF667-AS1 in (**a**) SKOV3 and (**b**) OVCAR3 cells transfected with miRNA mimics: c.el-67—cel-miR-67-3p, hsa-124—hsa-miR-124-3p, hsa-137—hsa-miR-137-3p. SKOV3: *p* (hsa-124 vs. mock) = 0.99, *p* (hsa-137 vs. mock) = 0.25, *p* (hsa-221 vs. mock) = 0.55; OVCAR3: *p* (hsa-124 vs. mock) = 0.057, *p* (hsa-137 vs. mock) = 0.99, *p* (hsa-221 vs. mock) = 0.99. Data are presented as the median and 25–75% percentiles (n = 4).

As can be seen from Figure 13, the effect of hsa-miR-137-3p on the level of ZNF667-AS1 was similar in the two OC cell lines.

**Figure 14:**
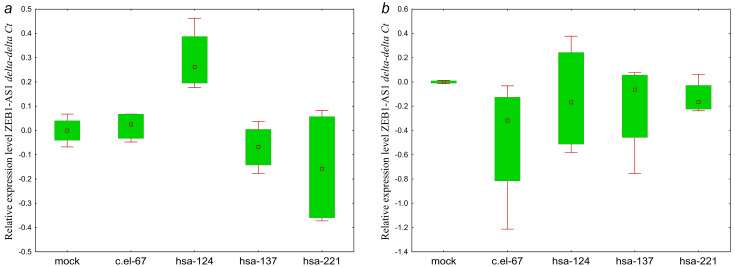
Changes in the levels of lncRNA ZEB1-AS1 in (**a**) SKOV3 and (**b**) OVCAR3 cells transfected with miRNA mimics: c.el-67—cel-miR-67-3p, hsa-124—hsa-miR-124-3p, hsa-137—hsa-miR-137-3p. SKOV3: *p* (hsa-124 vs. mock) = 0.99, *p* (hsa-137 vs. mock) = 0.99, *p* (hsa-221 vs. mock) = 0.72, OVCAR3: *p* (hsa-124 vs. mock) = 0.99, *p* (hsa-137 vs. mock) = 0.99, *p* (hsa-221 vs. mock) = 0.99. Data are presented as the median and 25–75% percentiles (n = 4).

Direct influence of hsa-miR-124-3p on the expression level of ZEB1-AS1 is absent in both cell lines (Figure 14). Here, we observed the opposite direction of the effects on lncRNA ZEB1-AS1 of hsa-miR-124-3p and hsa-miR-137-3p, which previously demonstrated a similar pattern of influence on targets. It should be noted that the effects mediated by hsa-miR-221 rather differed from hsa-miR-124-3p and hsa-miR-137-3p.

We also tested all studied lncRNAs for possible cross-regulation by microRNAs hsa-miR-124-3p and hsa-miR-137-3p.

**Figure 15:**
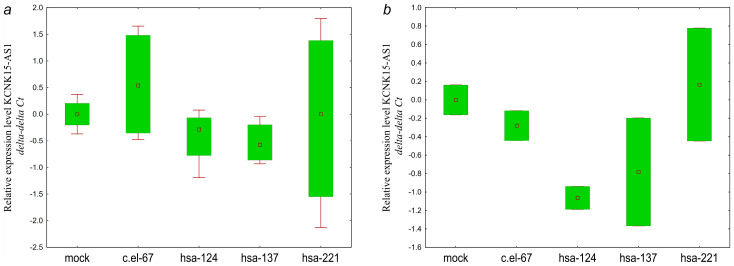
Changes in the levels of lncRNA KCNK15-AS1 in (**a**) SKOV3 and (**b**) OVCAR3 cells transfected with miRNA mimics: c.el-67—cel-miR-67-3p, hsa-124—hsa-miR-124-3p, hsa-137—hsa-miR-137-3p. SKOV3: *p* (hsa-124 vs. mock) = 0.99, *p* (hsa-137 vs. mock) = 0.78, *p* (hsa-221 vs. mock) = 0.10; OVCAR3: *p* (hsa-124 vs. mock) = 0.69, *p* (hsa-137 vs. mock) = 0.99, *p* (hsa-221 vs. mock) = 0.99. Data are presented as the median and 25–75% percentiles (n = 4).

Of all the lncRNAs, only KCNK15-AS1 showed a similar pattern of expression decrease in mimic-transfected cell lines (Figure 15).

Thus, GAS5 and ZNF667-AS1 showed expression changes generally consistent with the bioinformatic prediction in the hsa-miR-137-3p/GAS5 and hsa-miR-137-3p/ZNF667-AS1 pairs. However, the predicted regulatory link was not confirmed for the hsa-miR-124-3p/ZEB1-AS1 pair.

We were unable to investigate the regulation of lncRNAs MEG3, SEMA3B, SSTR5-AS1, HAND2-AS1, and MAGI2-AS3 by mimics because these lncRNAs were undetectable in these cells. This is consistent with the results of the abundance of these lncRNAs Affymetrix data compared to, for example, the more abundant GAS5 and ZNF667-AS1.

We compared the abundance of the studied lncRNAs using whole transcriptome RNA-seq data (EBI gene expression atlas) and our Affymetrix chip-based data. As can be seen from Table 7, the high-throughput methods were consistent with the RT-qPCR data regarding basal levels of lncRNA.

Therefore, using transfection of SKOV3 and OVCAR3 cells with miRNA mimics and the application of Affymetrix high-throughput chips in combination with bioinformatics data mining and analysis of EOC clinical samples, we identified six novel regulatory interactions: miR-137/GAS5-/KCNK15-AS1-/ZNF667-AS1 and miR-124/GAS5-/KCNK15-AS1-/ZNF667-AS1. Although these results require confirmation by direct binding assays, they provide important insights into the modes of regulatory activity of three lncRNAs in EOC: GAS5, KCNK15-AS1, ZNF667-AS1.

### 2.9. Prognostic Significance of the Studied Hypermethylated lncRNA Genes

**Figure 16:**
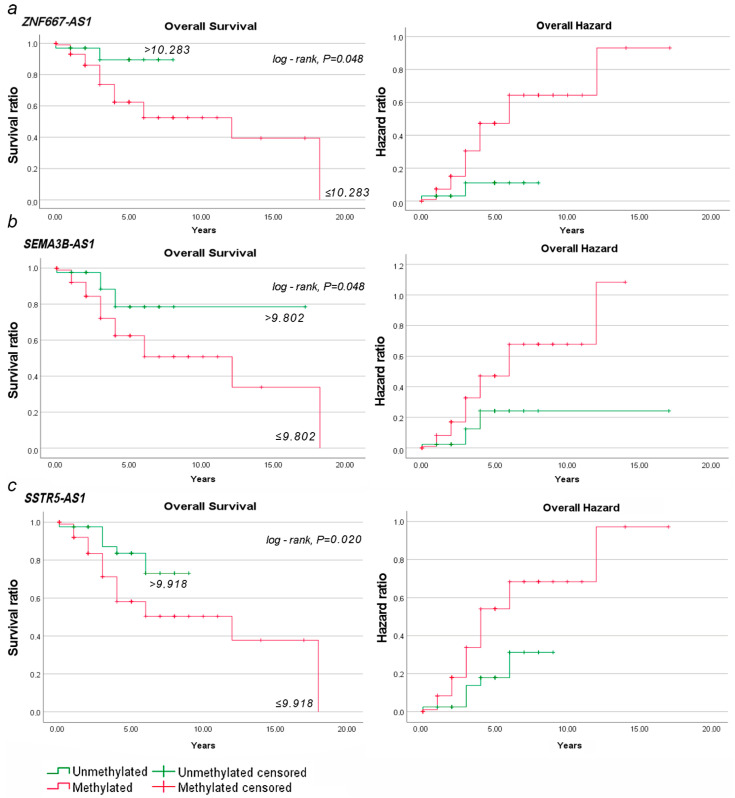
Analysis of overall survival and overall hazard in OC patients based on methylation status of (**a**) *ZNF667-AS1*, (**b**) *SEMA3B-AS1*, and (**c**) *SSTR5-AS1* genes.

We conducted a survival analysis of 140 OC patients (37 died and 103 alive): tumors with hypermethylation of the lncRNA genes *ZNF667-AS1*, *SEMA3B-AS1*, and *SSTR5-AS1* compared to tumors without hypermethylation of these loci. According to our results, after 10 years, 50% of patients with hypermethylation of *ZNF667-AS1* (Figure 16a) or *SEMA3B-AS1* (Figure 16b) or *SSTR5-AS1* (Figure 16c) genes experienced an increased risk of adverse outcomes (*p* = 0.048, 0.048, and 0.020, respectively).

Cumulative risk of triple factors was assessed by Cox regression analysis. Because of the lack of statistical power, we obtained a trend (*p* = 0.058) of cumulative risk of adverse outcomes. Thus, hypermethylation of the *ZNF667-AS1*, *SEMA3B-AS1*, and *SSTR5-AS1* genes may be a marker of reduced survival and poor outcome.

In addition, a panel of metastasis prognosis markers was developed based on six hypermethylated lncRNA genes (*GAS5*, *KCNK15-AS1*, *MEG3*, *SEMA3B-AS1*, *ZEB1-AS1*, and *ZNF667-AS1*). Detection of methylation of four panel markers allowed predicting EOC metastasis with high sensitivity (0.978), specificity (0.833), and reliability (AUC = 0.786, *p* < 0.0001).

It is noteworthy that two survival markers—*ZNF667-AS1* and *SEMA3B-AS1*—are undoubtedly of clinical significance, since they both also showed the most statistically significant (*p* < 0.001) increase in methylation level in EOC progression, including peritoneal metastases, metastases in total, advanced stages (III–IV), larger tumor size and extent (T3), and a higher histological grade of malignancy (G3–G4).

## 3. Discussion

The discovery of a new type of ncRNA, namely lncRNA, in the last two decades has revealed the incredible complexity of gene and genome expression regulation and the involvement of multiple regulatory layers. In this study, we investigated the regulatory properties of ten potentially hypermethylated lncRNA genes (GAS5, HAND2-AS1, KCNK15-AS1, MAGI2-AS3, MEG3, SEMA3B-AS1, SNHG6, SSTR5-AS1, ZEB1-AS1, and ZNF667-AS1) that were selected by analyzing genome-wide bisulfite methylation datasets (NCBI GEO, GSE81228 and GSE146556) for OC patients. In the set of 140 ovarian tumors and 123 histologically normal tissues, we demonstrated statistically significant (*p* < 0.0001) hypermethylation of all ten lncRNA genes. Moreover, a representative set of clinical samples allowed us to trace their association with EOC progression. Thus, we found a further statistically significant (*p* < 0.0001) increase in methylation levels of four genes (KCNK15-AS1, MEG3, SEMA3B-AS1, and ZNF667-AS1) at later clinical stages (III–IV vs. I–II) and in tumors of increased size or extent (T3 vs. T1–T2). In tumors of advanced histological grade (G3–G4 vs. G1–G2), the methylation level was statistically significantly (*p* < 0.001) increased for the *SEMA3B-AS1*, *SNHG6*, and *ZNF667-AS1* genes.

Next, using RT-qPCR and the subset of 90 samples, we showed that an increase in the methylation level corresponded to a decrease in the expression level. For all ten genes we identified a statistically significant (*r_s_* < −0.5, *p* < 0.001) negative correlation between changes in methylation and expression levels that indicated the functional significance of hypermethylation in the downregulation of ten lncRNA genes in EOC.

Our results on hypermethylation and downregulation of a number of lncRNA genes and their association with cancer progression obtained in a representative set of clinical EOC samples are consistent with the literature data, which were obtained mainly for OC cell cultures or tumors from other locations. Thus, for the lncRNA HAND2-AS1, inactivating gene hypermethylation, tumor suppressor activity, and the ability to increase sensitivity to antitumor therapy were shown in EOC cells [22,30], and ability to suppress invasion was demonstrated in endometrial carcinoma [31]. Gene hypermethylation, downregulation, and tumor-suppressor features were found for the lncRNA KCNKI5-ASI in gastric cancer [32]. Among the most proven tumor suppressor lncRNAs are MAGI2-AS3 and MEG3, which suppress OC cell proliferation and invasion or angiogenesis [21,33,34,35,36,37]. SEMA3B-AS1 was shown to exhibit tumor-suppressor properties and inhibition of breast cancer progression [38,39], as well as association with metastasis and prognosis of gastric cancer [40]. There is evidence of hypermethylation and downregulation of lncRNA SSTR5-AS1 and tumor-suppressive and anti-metastatic behavior of this lncRNA in laryngeal squamous cell carcinoma [41]. ZEB1-AS1 in OC was shown to have tumor-suppressor properties and the ability to restore chemosensitivity to PTX- and DDP-resistant A2780/R OC cells and to silence MMP19 in ZEB1-AS1-overexpressing cells [42]. The *ZNF667-AS1* (*MORT*) lncRNA gene was found hypermethylated and downregulated in ovarian and other cancers, and tumor-suppressive function of lncRNA ZNF667-AS1 was shown [19,43,44]. Inactivating hypermethylation of the *GAS5* gene and the tumor-suppressive function of this lncRNA have been previously reported in gastric, cervical, and other tumors, as well as by us in EOC [19,45,46].

At the same time, it should be emphasized that inactivating hypermethylation of the *KCNKI5-ASI*, *MAGI2-AS3*, *SSTR5-AS1*, and *ZEB1-AS1* genes in EOC was established by us for the first time, and these data confirm their tumor-suppressive nature.

Given the importance of intraperitoneal dissemination as a major route of metastasis in EOC [5,6,7], we associated hypermethylation of ten lncRNA genes with all types of metastases, including distant, lymph node, peritoneal, and greater omentum. We found that different ways of EOC metastasis were characterized by methylation of different lncRNA genes. Thus, methylation of the *HAND2-AS1* gene was mainly associated with lymphogenous metastasis, *KCNK15-AS1*, *MEG3*, and *SEMA3B-AS1* were specific for spreading through the peritoneum, and *MEG3*, *SEMA3B-AS1*, and *ZNF667-AS1* were specific for metastasis to the greater omentum. Moreover, a set of highly statistically significant (*p* < 0.0001) marker genes for the prognosis of EOC metastases of any types was determined: *KCNK15-AS1*, *MEG3*, *SEMA3B-AS1*, and *ZNF667-AS1* (see Figure 3). Interestingly, the same set of four lncRNA genes was statistically significantly (*p* < 0.0001) associated with advanced clinical stages, as well as tumor size and extent.

It should be noted that such a differentiated analysis of different types of metastases in EOC and the role of lncRNA genes in them has not been previously conducted by other authors, at least for these five lncRNAs: HAND2-AS1, KCNK15-AS1, MEG3, SEMA3B-AS1, and ZNF667-AS1.

Further, an interesting phenomenon was discovered: six lncRNA genes (*HAND2-AS1*, *MEG3*, *SEMA3B-AS1*, *SSTR5-AS1*, *ZEB1-AS1*, and *ZNF667-AS1*) showed a statistically significant (*p*~0.01–0.0001) decrease in methylation level in 59 macroscopic peritoneal metastases compared to the primary tumors of the same EOC patients, although five of these genes showed a statistically significant increase in methylation level in primary tumors of patients with metastases compared to tumors of patients without metastases. We hypothesized that the decreased methylation level of these lncRNA genes in macroscopic metastases may be due to the enhancement of mesenchymal properties in metastatic cells to enhance their motility.

Indeed, measurement of mRNA levels of five EMT markers (CDH1, VIM, SNAI2, ZEB1, and ZEB2 mRNAs) showed a statistically significant decrease (*p* < 0.05) in the level of the epithelial marker, CDH1 (E-cadherin) mRNA. Thus, it appears that in metastatic cells that have separated from the primary tumor and migrated into the abdominal cavity, a partial transition of EMT occurs, which is accompanied by a decrease in the methylation level of five lncRNA genes: *HAND2-AS1*, *KCNK15-AS1*, *MEG3*, *SEMA3B-AS1*, and *ZNF667-AS1*.

Over the last two decades, the role of partial EMT in cancer metastasis has been widely discussed [47,48]. Transition between epithelial and mesenchymal states ensures the acquisition of a motile mesenchymal phenotype by tumor cells, but in order for tumor cells to be able to switch from one motility mode to another depending on the cellular context and environmental conditions, they require migratory plasticity [15,49]. In classical EMT, for example, during embryonic development, epithelial cells lose all features of their epithelial origin and acquire a completely mesenchymal phenotype, known as full EMT, which is characterized by the so-called cadherin switch. During metastasis, cancer cells that originate from epithelial cells exhibit both mesenchymal and epithelial characteristics, corresponding to a hybrid E/M phenotype, indicating partial EMT [50,51]. Moreover, multiple tumor subpopulations associated with different stages of EMT were described: from epithelial to fully mesenchymal states, passing through many intermediate hybrid states [49]. The role of partial EMT in the dynamics and plasticity of tumor progression processes on many levels, as a multi-tool in orchestrating a complex of processes associated with metastasis, changes in tumor cell stemness, and an increase in their drug resistance, were noted [51,52]. These phenotypic changes are regulated by extracellular matrix components, exosomes, and EMT-related transcription factors [15,51]. Recent studies using single-cell sequencing techniques identified multiple double negative feedback loops involving EMT transcription factors. These feedback loops between EMT drivers and MET drivers fine-tune the cell’s EMT transition state [53].

Thus, the statistically significant decrease (*p* = 0.005) in the E-cadherin mRNA level observed by us in macroscopic metastases indicates partial EMT accompanied by a decrease in the methylation level of five lncRNA genes: *HAND2-AS1*, *KCNK15-AS1*, *MEG3*, *SEMA3B-AS1*, and *ZNF667-AS1*. Moreover, analysis of lncRNA expression in 31 PM samples compared to 31 initial primary tumors showed an increase in the expression level of lncRNAs HAND2-AS1 and MEG3 (see Figure 8c) in peritoneal metastases, which is consistent with a decrease in their methylation level. Moreover, the transition from the primary tumor to metastasis may include a number of events and processes, starting from the separation of metastatic cells from the primary tumor, movement, including within the ascetic fluid, and attachment to the peritoneal walls, completing the colonization of metastasis. It is likely that a series of processes involving changes in the epithelial status of cells, including partial EMT and partial reversion of EMT–MET, occur between the primary tumor and the colonized metastasis, since the functional roles of pEMT or pMET states can plastically vary depending on the state of dissemination and the extent of metastatic colonization [6,51,54]. Thus, further detailed studies of the change in the states of metastatic cells are required.

On the other hand, our bioinformatic screening of genes co-expressed with metastasis-associated lncRNAs (HAND2-AS1, KCNK15-AS1, MEG3, SEMA3B-AS1, and ZNF667-AS1) showed that they included a significant proportion of genes associated with EMT (see Table 1). Identified by us, hypermethylation and downregulation in tumors and metastases and bioinformatic prediction of EMT-associated target genes for five lncRNAs (HAND2-AS1, KCNK15-AS1, MEG3, SEMA3B-AS1, and ZNF667-AS1) are consistent with the literature data on their tumor-suppressive and antimetastatic functions and their association with EMT. Thus, for MEG3, the connection with EMT was shown in tumors of various localizations, including OC [55,56,57]. It was also reported that among the EMT-linked genes regulated by MEG3, many contain MEG3 binding sites [56]. KCNK15-AS1 suppresses cell proliferation, migration, and EMT in pancreatic cancer by regulating KCNK15 and PTEN [58]. SEMA3B-AS1 inhibits the invasion, proliferation, migration, and EMT of gastric cancer cells in vitro [40]. LncRNA ZNF667-AS1 suppresses the EMT process in oral and laryngeal squamous cell carcinoma [59,60]. MAGI2-AS3 inhibits bladder and pancreatic cancer progression through regulating EMT [61,62].

However, the effects of methylation of *HAND2-AS1*, *KCNK15-AS1*, *SEMA3B-AS1*, and *ZNF667-AS1* genes on metastasis and EMT in OC have not been previously reported and were identified here for the first time.

Next, for lncRNAs HAND2-AS1 and MAGI2-AS3, which were identified to have the highest number of potential target mRNAs (see Table 1), mRNAs that had regions of complementarity in addition to positive correlations were selected (see Table 2). Among the candidate genes, SERPINF1 mRNA (or otherwise, pigment epithelial factor, PEDF), which was predicted to have the highest affinity for lncRNAs HAND2-AS1 and MAGI2-AS3, as well as FKBP14 mRNA (FKBP prolyl isomerase 14), which was predicted to have affinity for MAGI2-AS3, although an order of magnitude lower affinity for HAND2-AS1 (see Table 2), were selected. Experimental data were obtained confirming the possibility of activating interaction in the pairs HAND2-AS1/SERPINF1, MAGI2-AS3/SERPINF1, and MAGI2-AS3/FKBP14, although the possibility of direct binding requires the use of special methods, such as the luciferase test, etc. As already noted, the lncRNA MAGI2-AS3 showed multifaced properties here, since SERPINF1 belongs to tumor suppressors that inhibit angiogenesis and metastasis [63,64] and FKBP14, on the contrary, belongs to endoplasmic reticulum proteins and is characterized by oncogenic behavior in tumors of different localizations [24,65].

The most proven mechanism of gene regulation involving lncRNA is the miRNA-mediated ceRNA model, according to which mRNA and lncRNA are endogenous RNAs competing for miRNA binding via the lncRNA/miRNA/mRNA axis [12,17]. An integrated approach using predictive analysis of NCBI datasets, experimental analysis of lncRNA and miRNA expression in clinical EOC samples, transfection of SCOV3 and OVCAR3 cells with miRNA mimics, and Affymetrix high-throughput chips allowed us to identify novel regulatory interactions: miR-137/GAS5-/KCNK15-AS1-/ZNF667-AS1 and miR-124/GAS5-/KCNK15-AS1-/ZNF667-AS1.

It should be also noted that the suppressor and regulatory functions of GAS5 via miRNA in cancer were widely demonstrated, in particular in OC reports concerning five different miRNAs, for example, miR-21 and miR-23a [66,67]. However, interactions of GAS5 with neither miR-137 nor miR-124 have not been previously reported. Regarding KCNK15-AS1, the role of this lncRNA not only in EOC but also in tumors in general was very little studied, and only its potential binding to miR-202 and miR-370 in lung cancer is known [68]. Neither ZNF667-AS1 interactions with miRNA in EOC nor potential interactions of ZNF667-AS1 with miR-124 or miR-137 in any cancer type were previously reported. However, a number of miRNAs were identified in tumors of some locations that may be associated with ZNF667-AS1, and, for example, in cervical cancer, ZNF667-AS1 suppresses progression via interaction with miR-93-3p [69]. Therefore, the potential interactions of lncRNAs GAS5, KCNK15-AS1, and ZNF667-AS1 with miR-124 and miR-137 that we found are of priority.

Kaplan–Meier analysis of survival data showed the prognostic potential of hypermethylation of the *SEMA3B-AS1*, *SSTR5-AS1*, and *ZNF667-AS1* genes. The prognostic significance of these three lncRNAs was shown in several cancer types, such as ZNF667-AS1 in glioma [70] and SEMA3B-AS1 and SSTR5-AS1 in esophageal cancer [71,72], but we report for the first time the prognostic potential of these three lncRNAs as overall survival markers in EOC. Recently, an article has been published reporting that a meta-analysis of 37 studies identified a panel of prognostic markers of overall survival of OC patients based on 42 non-coding RNAs (15 miRNAs, 24 lncRNAs, and 3 circular RNAs) with a hazard ratio of 1.39 (*p* = 0.32) [73]. We believe that our data will also be useful for selecting the most reliable risk factors.

In conclusion, in this work, the role of ten hypermethylated lncRNAs in the occurrence, progression, and metastasis of EOC was determined. Five genes associated with lymph node metastasis (*HAND2-AS1*) and peritoneal and omental metastasis (*KCNK15-AS1*, *MEG3*, *SEMA3B-AS1*, and *ZNF667-AS1*) were identified. For metastasis-associated lncRNA genes, data were obtained on their involvement in the processes of changing the epithelial–mesenchymal characteristics of cells during the transition from primary tumors to colonized macroscopic metastases in the abdominal cavity. These changes were described by partial EMT; however, apparently, they correspond to several intermediate states of metastatic cells, which requires more in-depth studies. Interestingly, four out of five metastasis-associated genes (*KCNK15-AS1, MEG3*, *SEMA3B-AS1*, and *ZNF667-AS1*), the methylation of which increased in primary tumors with intraperitoneal dissemination, were also associated with the clinical stages and size of tumors in patients, demonstrating the importance of peritoneal spread.

**Figure 17:**
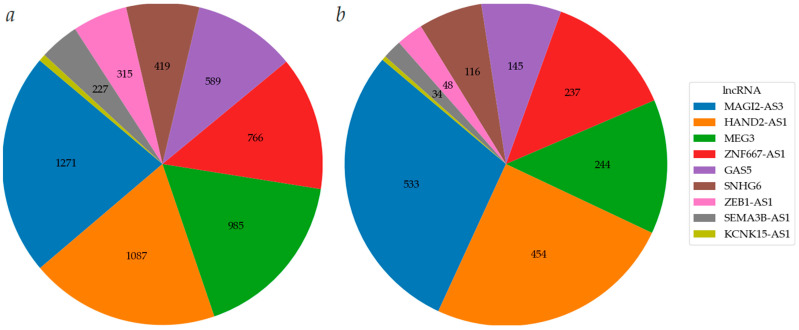
Comparison of the functional significance of the studied lncRNAs; (**a**) potential mRNA targets of nine lncRNAs according to the analysis of co-expressed mRNAs at *r_s_* > 0.4 in the GSE211669 dataset; KCNK15-AS1 had 41 target mRNAs; (**b**) EMT-associated genes among the identified mRNA targets according to GeneCards; 8 of 41 target mRNAs for KCNK15-AS1 were EMT-associated.

Our results, in particular the bioinformatic screening of co-expressed mRNAs in EOC, allowed us to compare the functional activity of all ten lncRNAs, for example, by the total number of potential direct or indirect mRNA targets (Figure 17).

It turned out that the following lncRNAs were most actively involved in gene regulation through direct or indirect mRNA activation: HAND2-AS1, MAGI2-AS3, MEG3, and ZNF667-AS1 (Figure 17a). The same lncRNAs were most associated with EMT genes (Figure 17b). LncRNAs GAS5, SNHG6, ZEB1-AS1, SEMA3B-AS1, and KCNK15-AS1 had several times fewer potential targets. SSTR5-AS1 did not have any mRNA targets with *r_s_* > 0.4, and it is possible that its participation in the pathogenesis of EOC is realized by a different mechanism. According to our data, hypermethylation and decreased expression of all ten studied lncRNAs are involved in the development of EOC, and inactivating methylation of HAND2-AS1, MEG3, ZNF667-AS1, SEMA3B-AS1, and KCNK15-AS1 is most involved in EOC progression and metastasis, although KCNK15-AS1 has less potential to activate mRNA of protein-coding genes.

In addition to the analysis of prognostic databases and clinical samples, transfection of two cell lines in combination with Affymetrix chips coupled with RT-qPCR were used to assess potential interactions of any of the ten lncRNAs with the most promising predicted miRNAs. We successfully identified potential interactions of miR-137/GAS5-/KCNK15-AS1-/ZNF667-AS1 and miR-124/GAS5-/KCNK15-AS1-/ZNF667-AS1, likely involving direct binding in EOC.

Furthermore, we demonstrated the prognostic potential of poor survival for hypermethylation of the *ZNF667-AS1, SEMA3B-AS1*, and *SSTR5-AS1* genes in EOC. Moreover, the association of methylation of lncRNA genes *SEMA3B-AS1* and *ZNF667-AS1* with all indicators of EOC progression (clinical stage, tumor extent, histological grade, metastasis, and overall survival) is also a clinically significant result.

## 4. Materials and Methods

### 4.1. Bioinformatics

Data from whole-genome bisulfite sequencing of OC samples from NCBI Gene Expression Omnibus (NCBI GEO) were used as the primary test for screening hypermethylated lncRNA genes in ovarian tumors. Two datasets were used: GSE81228 (https://www.ncbi.nlm.nih.gov/geo/query/acc.cgi?acc=GSE81228, accessed on 1 August 2024), and GSE146556 (https://www.ncbi.nlm.nih.gov/geo/query/acc.cgi?acc=GSE146555, accessed on 1 August 2024), which included data for 17 (13 and 4, respectively) patients with high-grade serous OC with normal ovarian surface epithelial cells and fallopian tube epithelial cells as control samples.

To assess the predicted changes in expression levels of the studied lncRNA genes in ovarian tumors, the GEPIA 2.0 database (Gene Expression Profiling Interactive Analysis, http://gepia2.cancer-pku.cn/#index, accessed on 1 August 2024), which included data for 426 tumor samples and 88 normal ovarian tissues, was used [74].

Evaluation of predicted mRNAs positively or negatively correlated with lncRNAs was performed in R using the dataset from NCBI GEO (https://www.ncbi.nlm.nih.gov/geo/query/acc.cgi?acc=GSE211669, accessed on 1 August 2024) for 131 patients of high-grade serous OC; data were taken into account at *r_s_* > 0.4, *p* < 10^−8^.

The assessment of predicted miRNAs negatively correlated with the studied lncRNAs was performed using the GSE119055 dataset (https://www.ncbi.nlm.nih.gov/geo/query/acc.cgi?acc=GSE119055, accessed on 1 August 2024), containing data for 6 OC samples and 3 normal ovarian tissue samples; data were taken into account at *r_s_* < −0.6, *p* < 0.0025.

Local sequence alignment between miRNAs and lncRNAs as well as between mRNAs and lncRNAs was performed using the NCBI nucleotide archive and the Smith–Waterman algorithm (https://www.sciencedirect.com/science/article/abs/pii/0022283681900875?via%3Dihub, accessed on 1 August 2024) [75].

Genes associated with EMT were selected using the GeneCards (https://www.genecards.org/Search/Keyword?queryString=epithelial-mesenchymal, accessed on 1 August 2024) and dbEMT 2.0 (http://dbemt.bioinfo-minzhao.org/, accessed on 1 August 2024) databases.

All databases used are summarized in Supplementary Table S24.

### 4.2. Tissue Samples

Ovarian tumor samples were collected and characterized morphologically and clinically at the N.N. Blokhin National Medical Research Center of Oncology (Moscow, Russia). This study adhered to the principles of voluntariness and confidentiality outlined in the World Medical Association’s Declaration of Helsinki [76], with informed consent obtained from all patients. The samples were collected in accordance with the guidelines issued by the Ethics Committee of the N.N. Blokhin National Medical Research Center of Oncology and in cooperation with them. Tumor tissues and matched histologically normal tissues were obtained from the patients after surgical resection prior to radiation or chemotherapy and were stored in liquid nitrogen. Diagnoses were verified by histopathology, and only the samples containing 50–70% or more tumor cells were used in the studies. Matched controls were histologically confirmed to be normal epithelial cells. The tumor samples were characterized based on the tumor-node-metastasis according to the International System of Classification of Tumors, according to the staging classification of the Union for International Cancer Control [77], and using the criteria for classification developed by the World Health Organization (WHO) [78]. The clinical and morphological characteristics of the samples are presented in Table 8 and Supplementary Table S25.

Most samples (76%, 106/140) were serous ovarian adenocarcinomas. The set of samples included 140 primary ovarian tumors (without and with metastases), 123 matched histologically normal ovarian tissues, 59 peritoneal metastases (PM), and 18 ovarian tissue samples from post-mortal women without any cancer in their anamnesis. The whole set of samples was used in the DNA methylation studies. Tissue samples were stored at −70 °C. The frozen tissues were homogenized using a TissueRuptor II homogenizer-dispersant (Qiagen, Hilden, Germany) in liquid nitrogen.

### 4.3. DNA and Total RNA Isolation and Reverse Transcription

DNA from the tissues was isolated using phenol extraction as per standard protocols. Total RNA was isolated using a guanidinium thiocyanate-phenol-chloroform extraction protocol [79]. Total RNA from cell lines was isolated using TRIzol (Thermo Fisher Scientific, Waltham, MA, USA, #15596018) following the manufacturer’s instructions but with the addition of an extra chloroform extraction step to increase RNA purity.

Before use, all RNA samples were treated with RNase-free DNase I (Thermo Fisher Scientific, #EN0521) according to the manufacturer’s protocol. RNA concentration and purity were determined spectrophotometrically using NanoDrop ND-1000 (Thermo Fisher Scientific): evaluation of absorbance at 260 nm and the ratios of absorbance at 260 nm/230 nm and 260 nm/280 nm, respectively. The quality and concentration of DNA were also evaluated using the NanoDrop spectrophotometer (Thermo Fisher Scientific). RNA integrity was estimated by the ratio of band intensities for 28S rRNA vs. 18S rRNA or by RNA Integrity Number (RIN) obtained from microfluidic capillary electrophoresis performed on an Bioanalyzer 2100 (Agilent Technologies, Santa Clara, CA, USA) with an RNA 6000 Nano kit (Agilent Technologies, #5067-1511). The 28S and 18S rRNA band intensities were estimated via electrophoresis in a 2% agarose gel using the Sub-Cell GT Horizontal Electrophoresis System (Bio-Rad, Hercules, CA, USA) followed by gel imaging with the Gel Doc XR+ Gel Documentation System (Bio-Rad). The RNA was considered acceptable for further use if the bands of the 28S and 18S rRNAs had an intensity ratio of about 2:1, and the A260/A280 ratio was in the range 1.8–2.1. All cDNA was synthesized from 1 μg of total RNA using the M-MLV reverse transcriptase and random specific set of oligonucleotides according to the manufacturer’s protocol (Thermo Fisher Scientific, #28025021 and #AM5722G). One microgram of each RNA sample extracted from cell lines was then reverse-transcribed using random hexamer or nanomer primers with M-MLV reverse transcriptase as a part of the RevertAid H Minus First Strand cDNA Synthesis Kit (Thermo Fisher Scientific, #K1632) following the manufacturer’s instructions.

### 4.4. Quantitative PCR (qPCR) for Expression Analysis

The levels of ten lncRNAs and seven mRNAs were assessed by qPCR using SYBR Green/ROX qPCR Master Mix (Thermo Fisher Scientific, #K0222) on a CFX96 Real-Time PCR Detection System (Bio-Rad) with the primers and PCR conditions given in Supplementary Table S26. *B2M* was used as a reference gene [80]. All PCR reactions were performed in triplicate, and each assay included negative control reactions that lacked cDNA.

Relative quantification according to the ΔΔCt-method [81] was used for data analysis. Considering expression level variability of reference genes and estimated errors, less than twofold changes (ΔΔCt ≤ 1) in mRNA, miRNA, or lncRNA levels were considered as retentions. For transfected cells, normalization was performed to the expression of genes in the cells incubated with lipofectamin 2000 only (mock).

MiRNA expression levels were analyzed by qPCR using TaqMan MicroRNA Assays (Thermo Fisher Scientific): hsa-miR-124-3p (Assay ID: 4427975-001182), hsa-miR-124-5p (Assay ID: 4427975-002197), hsa-miR-137-3p (Assay ID: 4427975-001129), hsa-miR-33b-5p (Assay ID: 4427975-002085); RNU48 (Assay ID: 4427975-001006) and RNU6 (Assay ID: 4427975-001093) expression levels were used as references.

The investigation of expression level changes for ten lncRNAs was performed in the set of 90 paired T/N EOC samples and 31 PM samples, four miRNAs were analyzed in 41 paired T/N EOC samples; mRNA levels of five EMT markers (CDH1, SNAI2/SLUG, ZEB1, ZEB2, VIM) were evaluated in 46 paired T/N EOC samples and 30 paired PM/N samples, and two other mRNAs (FKBP14 and SERPINF1)—in 44 paired (27 T/N + 17 PM/N) EOC samples.

### 4.5. Quantitative Methylation-Specific PCR (qMSP)

Bisulfite DNA conversion and qMSP were performed as previously reported [82]. Briefly, the Epitect Fast DNA bisulfite kit (Qiagen, #59826) was used for bisulfite conversion of DNA. The qPCRmix-HS SYBR reagent kit (Evrogen, Moscow, Russia, #PK147L) and CFX96 Real-Time PCR Detection System (Bio-Rad) were used. The primers designed for qMSP are listed in Supplementary Table S26. The completeness of DNA conversion was determined using the *ACTB* control locus with oligonucleotides specific to the unconverted matrix. For comparative analysis of amplification efficiency, the *ACTB* locus was also used with oligonucleotides specific to the converted matrix. Commercial DNA preparation #G1471 (Promega, Madison, WI, USA) served as a control for unmethylated alleles, while commercial DNA preparation #SD1131 (Thermo Fisher Scientific) was used as a positive control for 100% methylation. Oligonucleotide sequences and PCR conditions for lncRNA genes are shown in Supplementary Table S27.

### 4.6. Cell Lines and Culture Conditions

SK-OV-3 (SKOV-3, SKOV3, ATCC-HTB-77) were purchased from the Russian Cell Culture Collection maintained by the Institute of Cytology, Russian Academy of Sciences (St. Petersburg, Russia). Cell line characteristics, including karyotype analysis data, were provided by the vendor. The identity of the cell line was not authenticated further. The cells were cultured in Dulbecco’s modified Eagle’s medium (DMEM, Thermo Fisher Scientific) supplemented with 10% (*v*/*v*) fetal bovine serum (FBS) (Thermo Fisher Scientific), 1% non-essential amino acids, 50 µg/mL gentamicin (Thermo Fisher Scientific), 4 mM L-glutamine, and 4.5 g/L D-glucose in a humidified atmosphere containing 5% CO_2_ in an incubator (Sanyo, Moriguchi, Osaka, Japan) at 37 °C. The cells were subcultured at confluence by treatment with 0.05% trypsin and 0.02% EDTA in phosphate-buffered saline (PBS).

OVCAR-3 (OVCAR3, ATCC-HTB-161) were purchased from the ATCC (Manassas, VA, USA). Cell line characteristics, including karyotype analysis data, were provided by the vendor. The identity of the cell line was not authenticated further. The cells were cultured in RPMI-1640 (Thermo Fisher Scientific) supplemented with 10% (*v*/*v*) fetal bovine serum (FBS) (Thermo Fisher Scientific), 1% non-essential amino acids, 0.128 mg/mL human recombinant insulin (Merck, Darmstadt, Germany), 50 µg/mL gentamicin (Thermo Fisher Scientific), 4 mM L-glutamine, and 4.5 g/L D-glucose in a humidified atmosphere containing 5% CO_2_ in an incubator (Sanyo) at 37 °C. The cells were subcultured at confluence by treatment with 0.05% trypsin and 0.02% EDTA in phosphate-buffered saline (PBS).

### 4.7. RNA Duplexes and Transfection

Synthetic miRNAs were designed to mimic mature endogenous miRNAs. Sequences of hsa-miR-124-3p and its antisense strand were identical to the strands of RNA duplexes used, as described previously [83]. The duplexes corresponding to hsa-miR-137-3p and hsa-miR-221-3p were designed similarly to hsa-miR-124-3p by introducing 3′-overhangs and the thermodynamic destabilization of the relevant seed-containing end of the duplexes to facilitate activation of the sense strand. RNAs of each miRNA duplex were synthesized (DNK-Sintez, Moscow, Russia), resuspended at 200 μM, annealed by heating to 95 °C, and then slowly cooled to 37 °C. For transfection, 0.4 million cells were plated per well in a six-well plate supplied with 2 mL of medium (DMEM + 10% FBS). After 24 h of culture (at 50% to 80% confluence), the cells were supplied with fresh medium and transfected with an RNA duplex at a final amount of 100 pmol/well using GenJect-39 (Molecta, Moscow, Russia) and Opti-MEM (Thermo Fisher Scientific) following the manufacturer’s protocol. After 24 h, the cells were harvested and analyzed. Transfection of cel-miR-67-3p RNA duplexes exhibiting low homology with human miRNA sequences was used as a specificity control, where assessment of expression for hsa-miR-1-3p RNA duplexes together with its target twinfilin actin binding protein 1 (TWF1) [83,84].

At least 4 biological replicates were used when setting up the experiments. Technical replicates were also used when performing RT-qPCR.

### 4.8. Affymetrix Whole Transcriptome Gene Expression Analysis

A GeneChip HTA 2.0 Array System (Affymetrix, Santa Clara, CA, USA) was used for gene expression analysis. Biotinylated sense-strand DNA targets were prepared from 500 ng of total RNA using the GeneChip WT PLUS Reagent kit (Thermo Fisher Scientific). Hybridization, labeling, and washing were performed using the GeneChip Hybridization Wash and Stain Kit (Thermo Fisher Scientific) using Fluidics Station 450 (Affymetrix). The arrays were scanned using a Gene Scan 7G (Affymetrix) system. Standard Affymetrix quality control was conducted using the Transcriptome Analysis Console (Thermo Fisher Scientific) v.4.0.2.15.

### 4.9. Statistical Analysis

Statistical processing of the results was carried out using the package of statistical programs IBM SPSS Statistics 27, which included the determination of the median and interquartile range. The R software environment (version 4.1.1) was used to build heatmaps and correlation matrices. To assess the significance of differences, the nonparametric Mann–Whitney U was used. Spearman’s correlation analysis was also applied. Differences were considered statistically significant at *p* ≤ 0.05. A Benjamini–Hochberg correction for multiple comparisons was performed and false discovery rate (FDR) values were calculated.

For most patients, overall survival data were tracked for more than 10 years. Survival analysis depending on methylation levels of lncRNA genes was performed with the non-parametric Kaplan–Meier method using the IBM SPSS Statistics 27 statistical package with statistical significance of differences between groups assessed by the log-rank test. For assessing cumulative risk of triple factor, a Cox regression test was used in the same package.

## Figures and Tables

**Table 1:** Intersections between lists of mRNAs correlated with lncRNAs and lists of genes associated with EMT in EOC.

LncRNA	Correlated mRNAs *	Intersected EMT Genes by GeneCards **	Intersected EMT Genes by dbEMT 2.0 ***
HAND2-AS1	**1088** mRNAs *r_s_* > 0.4, *p* < 10^−5^	**455**, **42%**	**67**, **6.2%**: ACTA2, KIT, IL11, HIC1, SATB2, EPB41L3 FGF1, ERG, MRC2, MICAL2, CXCL14, MAP4K4, CYP7B1, FOXP2, NOG, CCND2, SEMA7A, LOXL3, MARVELD1, VTN, ACTG2, TLE4, TIMP3, SLIT3, SKI, SHC1, ATXN1, PTPRZ1, NKX3-2, ERRFI1, PLS3, PDGFRB, ACKR4, PDGFB, LIMA1, NOTCH4, MMP19, MMP13, MMP11, KDR, ID2, NR4A1, EHD2, GLS, GJB2, PLXND1, FBLN1, F2RL2, ETV1, ETS1, EMP3, ELK3, EDNRA, AGTR1, CYP1B1, CSPG4, VCAN, COL8A1, CNTN1, CTHRC1, VSIG4, FSTL1, FOXN3, PDPN, CDKN1A, CDH11, CDH5
KCNK15-AS1	**575** mRNAs *r_s_* > 0.3, *p* < 10^−3^	**141**, **24%**	**21**, **3.6%**: FHL1, PRKCQ, ELAVL1, SRI, SNW1, SUFU, SKP1, ARHGEF2, PCMT1, OLA1, CCNG2, SLC39A6, PAQR3, SERPINI1, ITGA2, CCNDBP1, BMI1, PRKCE, PTEN, ZBTB33, CDX2
	**41** mRNAs *r_s_* > 0.4, *p* < 10^−5^	**8**, **20%**	**1**, **2.4%**: SERPINI1
MAGI2-AS3	**1272** mRNAs *r_s_* > 0.4, *p* < 10^−5^	**534**, **42%**	**83**, **6.5%**: ACTA2, PTHLH, CDK14, SUFU, SERPINE1, KIT, ITGA2, IL11, TNC, HIC1, GLI2, EPB41L3, FGF1, ERG, CREB1, MRC2, MICAL2, CXCL14, RASAL2, MAP4K4, QKI, CYP7B1, FOXP2, KL, NOG, CCND2, AJUBA, SEMA7A, LOXL3, MARVELD1, SETD7, VSNL1, ACTG2, TLE4, TJP1, TIMP3, SLIT3, ST8SIA1, SHC1, CPEB1, ATXN1, RASA1, PTPRZ1, NKX3-2, PLS3, PDGFRB, ACKR4, PDGFB, LIMA1, ROR1, MMP19, MMP13, MMP11, MEF2D, SMAD9, KDR, IRS1, FAS, ID2, APBB1, EHD2, LAMA1, GLS, DKK3, PHLDA1, F2RL2, ETV1, ELK3, EDNRA, AGTR1, DAPK1, CSPG4, VCAN, COL8A2, COL8A1, CNTN1, CTHRC1, FSTL1, FOXN3, PDPN, CDH13, CDH11, CDH5
MEG3	**985** mRNAs *r_s_* > 0.4, *p* < 10^−5^	**245**, **25%**	**32**, **3.2%**: ACTA2, PKD1, KIT, IL11, HIC1, GLI2, SATB2, TET3, CCND2, SEMA7A, MZF1, VTN, TSC1, TBX3, PTPRZ1, PROX1, ERRFI1, PDGFRB, LIMA1, NOTCH4, MMP19, MDM4, NR4A1, FHOD1, LAMA1, KDM6B, F3, AGTR1, DAPK1, FSTL1, CDK3, CDH11
SEMA3B-AS1	**228** mRNAs *r_s_* > 0.4, *p* < 10^−5^	**35**, **15%**	**5**, **2.2%**: HRAS, NOX1, S100A6, EEF1D, MSLN
	**166** mRNAs *r_s_* < −0.4, *p* < 10^−5^	**27**, **16%**	**3**, **1.8%**: GJB1, FBXO11, LRG1
ZNF667-AS1	**767** mRNAs *r_s_* > 0.4, *p* < 10^−5^	**238**, **31%**	**25**, **3.3%**: RAF1, MTA3, PBXIP1, PFN2, PCBP1, SLC39A6, CREB1, ZFYVE9, HS6ST2, AJUBA, CUL3, NUBPL, ZNF143, TJP1, SIAH2, PIK3R1, ROR1, ITGA6, BIRC2, OLA1, NEDD4L, SNW1, ELK1, DVL2, KDM5B

Note: positive correlations were mainly scored, negative correlations were scored for SEMA3B-AS1 only. * The complete lists of mRNAs correlated with examined lncRNAs are given in Supplementary Tables S1–S11; ** lists of mRNAs intersected with EMT genes according to GeneCards (https://www.genecards.org/Search/Keyword?queryString=epithelial-mesenchymal, accessed on 1 August 2024) are given in Supplementary Tables S12–S20; *** (http://dbemt.bioinfo-minzhao.org/, accessed on 1 August 2024).

**Table 2:** Top 10 results of screening for potentially interacting mRNAs and lncRNAs in EOC.

No.	Pairs		Correlation Study		Local Sequence Alignment	
	mRNA	lncRNA	*r_s_*	*p*-Value	Number *	Proportion **
1	PROCR	MAGI2-AS3	0.42	4.5 × 10^−7^	32	0.026
2	**SERPINF1**	**HAND2-AS1**	0.65	7.2 × 10^−17^	7	0.025
3	TMEM30C	SEMA3B-AS1	0.41	1.5 × 10^−6^	8	0.025
4	**SERPINF1**	**MAGI2-AS3**	0.61	6.1 × 10^−15^	6	0.022
5	BBIP1	MEG3	0.41	8.9 × 10^−7^	42	0.020
6	**FKBP14**	**MAGI2-AS3**	0.59	1.5 × 10^−13^	23	0.018
6a	**FKBP14**	**HAND2-AS1**	0.60	3.6 × 10^−14^	9	0.007
7	FAS	MAGI2-AS3	0.43	2.1 × 10^−7^	12	0.018
8	EPC1	ZEB1-AS1	0.53	6.6 × 10^−11^	5	0.018
9	EPC1	ZNF667-AS1	0.43	3.0 × 10^−7^	5	0.018
10	COX7A1	HAND2-AS1	0.44	1.4 × 10^−7^	6	0.018

Note: lncRNAs and mRNAs studied experimentally are shown in bold; * number of nucleotides in complementary sites; ** proportion of complementary nucleotides in sites relative to the number of nucleotides in mRNA. 6a is given for comparison.

**Table 3:** Predicted lncRNA/miRNA interactions according to correlation analysis of the GSE119055 dataset and local sequence alignment.

miRNA	lncRNA	*r_s_*	*p*-Value	Complementarity Site
hsa-miR-124-5p	ZNF667-AS1	−0.61	0.0098	7mer-m8 g-bulged
hsa-miR-124-5p	MAGI2-AS3	−0.77	0.0003	6mer
hsa-miR-124-5p	SSTR5-AS1	−0.64	0.0057	6mer
hsa-miR-124-5p	GAS5	−0.71	0.0014	
hsa-miR-124-5p	KCNK15-AS1	−0.70	0.0018	
hsa-miR-124-5p	SNHG6	−0.64	0.0052	
hsa-miR-124-5p	ZEB1-AS1	−0.76	0.0004	
hsa-miR-124-3p	SSTR5-AS1	−0.68	0.0026	6mer
hsa-miR-124-3p	ZEB1-AS1	−0.65	0.0057	6mer
hsa-miR-137-3p	GAS5	−0.64	0.0060	7mer-m8 *
hsa-miR-137-3p	MEG3	−0.75	0.0006	7mer-m8
hsa-miR-137-3p	SEMA3B-AS1	−0.69	0.0021	
hsa-miR-137-3p	ZNF667-AS1	−0.65	0.0046	
hsa-miR-33b-5p	HAND2-AS1	−0.71	0.0014	8mer
hsa-miR-33b-5p	KCNK15-AS1	−0.77	0.0003	
hsa-miR-33b-5p	MAGI2-AS3	−0.63	0.0064	7mer-m8
hsa-miR-33b-5p	MEG3	−0.67	0.0034	
hsa-miR-33b-5p	SEMA3B-AS1	−0.65	0.0045	
hsa-miR-33b-5p	SNHG6	−0.67	0.0034	

Note: * predicted by the StarBase (https://rnasysu.com/encori/, accessed on 1 August 2024) and miRcode (http://www.mircode.org/, accessed on 1 August 2024) databases.

**Table 4:** Reduction in mRNA and lncRNA levels in response to transfection of SKOV3 cells with the miR-137-3p mimic.

Gene Symbol	Description	Fold Change	*p*-Value	FDR *p*-Value
**mRNA** (total scored—42, *p* < 0.3)				
*CNN2*	calponin 2	−1.63	**0.02**	1
*CHRNA6*	cholinergic receptor, nicotinic alpha 6	−1.06	0.29	1
**lncRNA** (total scored—102, *p* < 1)				
*HIF1A-AS2*	HIF1A antisense RNA 2	−1.57	**0.002**	1
*SNHG8*	small nucleolar RNA host gene 8	−1.14	0.10	1
***GAS5***	growth arrest-specific 5	−1.03	0.69	1
*PROX1-AS1*	PROX1 antisense RNA 1	−1.01	0.91	1
*LINC00293*	long intergenic non-protein coding RNA 293	−1.00	0.93	1

Note: GAS5 is marked in bold as the center of interest; *p* < 0.05 are shown in bold.

**Table 5:** Reduction in mRNA and lncRNA levels in response to transfection of OVCAR3 cells with the miR-137-3p mimic.

Gene Symbol	Description	Fold Change	*p*-Value	FDR *p*-Value
**mRNA** (total scored—28, *p* < 0.05)				
*CNN2*	calponin 2	−1.55	**0.0002**	0.37
*C2orf48*	chromosome 2 open reading frame 48	−1.18	**0.04**	0.56
**lncRNA** (total scored—34, *p* < 0.3)				
*SNHG8*	small nucleolar RNA host gene 8	−1.21	**0.02**	0.51
*HIF1A-AS2*	HIF1A antisense RNA 2	−1.28	0.10	0.66
***GAS5***	growth arrest-specific 5	−1.07	0.22	0.77
*HCG11*	HLA complex group 11 (non-protein coding)	−1.06	0.30	0.81

Note: GAS5 is marked in bold as the center of interest; *p* < 0.05 are shown in bold.

**Table 6:** Reduction in mRNA and lncRNA levels in response to transfection of SKOV3 cells with the miR-124-3p mimic.

Gene Symbol	Description	Fold Change	*p*-Value	FDR *p*-Value
**mRNA** (total scored—94, *p* < 0.3)				
*CD40*	CD40 molecule, TNF receptor superfamily member 5	−1.57	**0.0004**	0.11
*OPRD1*	opioid receptor, delta 1	−1.07	0.29	0.85
**lncRNA** (total scored—30, *p* < 0.3)				
*SNHG8*	small nucleolar RNA host gene 8	−1.60	**0.003**	0.25
***GAS5***	growth arrest-specific 5	−1.27	**0.01**	0.37
*C21orf91-OT1*	C21orf91 overlapping transcript 1	−1.07	0.28	0.84

Note: GAS5 is marked in bold as the center of interest; *p* < 0.05 are shown in bold.

**Table 7:** Comparison of lncRNA abundance in RNA-seq (the E-MTAB-2770 dataset) and Affymetrix HTA 2.0 data.

Gene Name	SKOV3, RNA-Seq	SKOV3, Affymetrix	OVCAR3, RNA-Seq	OVCAR3, Affymetrix
*GAPDH*	2174.0	18.2	2708.0	18.9
*GAS5*	98.0	7.7	319.0	13.5
*HAND2-AS1*		4.9	0.1	4.4
*KCNK15-AS1*	4.0	N/A	3.0	N/A
*MAGI2-AS3*	0.1	5.8	0.4	5.1
*MEG3*		5.2	N/A	4.6
*SEMA3B-AS1*	3.0	7.0	7.0	6.2
*SNHG6*	122.0	N/A	142.0	N/A
*SSTR5-AS1*	0.3	5.8	N/A	6.5
*ZEB1-AS1*	7.0	8.1	2.0	5.3
*ZNF667-AS1*	20.0	8.2	7.0	6.7

**Table 8:** Summary of clinical data for the sample set examined in this study.

Clinical and Histological Characteristics		N = 140	With PM N = 59
Histological type	Serous adenocarcinoma	106	48
	Endometrioid adenocarcinoma	21	7
	Mucinous adenocarcinoma	8	3
	Clear cell adenocarcinoma	4	1
	Undifferentiated carcinoma	1	0
Stage	I	25	3
	II	22	7
	III	78	37
	IV	15	12
Size	T1	27	4
	T2	24	9
	T3	89	46
Grade	G1	36	13
	G2	36	12
	G3	66	33
	G4	2	1
Lymph node metastases	N0	110	45
	N1–N2	30	14
Distant metastases	M0	125	47
	M1	15	12
Peritoneal metastases	Without	70	13
	With	70	46
Metastases to the great omentum	Without	70	17
	With	70	42
Ascite	Without	79	20
	With	61	39

Note: PM—peritoneal metastases.

## Data Availability

The original contributions presented in the study are included in the article/Supplementary Materials. Further inquiries can be directed to the corresponding author.

## Supplementary Materials

The following supporting information can be downloaded at: https://www.mdpi.com/article/10.3390/ijms252111843/s1.
