# Supplementary material for: Ten Hypermethylated lncRNA Genes Are Specifically Involved in the Initiation, Progression, and Lymphatic and Peritoneal Metastasis of Epithelial Ovarian Cancer
Source: Int J Mol Sci. 2024 Nov 4;25(21):11843. doi: 10.3390/ijms252111843 (PMC11547154; doi:10.3390/ijms252111843)
Supplement: Supplementary file 1 [file ijms-25-11843-s001.zip › Table S24_Databases_2024.10.27.pdf]

Supplementary Table S24. List of databases used in the study.

Name of database	Type of data retrieved	Direct link*
NCBI GEO, GSE81228, 13 T/N	hypermethylated lncRNA genes in OC	https://www.ncbi.nlm.nih.gov/geo/query/acc.cgi?acc=GSE81228
NCBI GEO, GSE146555, 4 T/N		https://www.ncbi.nlm.nih.gov/geo/query/acc.cgi?acc=GSE146555
GEPIA 2.0, 426 T, 88 N	lncRNA expression level changes in OC	http://gepia2.cancer-pku.cn/#index
NCBI GEO, GSE211669, 131 T/N	mRNAs correlated with lncRNAs in OC	https://www.ncbi.nlm.nih.gov/geo/query/acc.cgi?acc=GSE211669
NCBI GEO, GSE119055, 6 T, 4 N	miRNAs correlated with lncRNAs in OC	https://www.ncbi.nlm.nih.gov/geo/query/acc.cgi?acc=GSE119055
GeneCards	EMT-associated genes	https://www.genecards.org/Search/Keyword?queryString=epithelial-mesenchymal
dbEMT 2.0		http://dbemt.bioinfo-minzhao.org/
Expression Atlas, E-MTAB-2770	lncRNA abundance in RNA-Seq data	https://www.ebi.ac.uk/gxa/experiments/E-MTAB-2770/Results

Note: *Accessed on 1 August 2024; OC – ovarian cancer; T – tumor samples; N – normal samples; T/N – paired (tumor/normal) samples.
