# Supplementary material for: Ten Hypermethylated lncRNA Genes Are Specifically Involved in the Initiation, Progression, and Lymphatic and Peritoneal Metastasis of Epithelial Ovarian Cancer
Source: Int J Mol Sci. 2024 Nov 4;25(21):11843. doi: 10.3390/ijms252111843 (PMC11547154; doi:10.3390/ijms252111843)
Supplement: Supplementary file 1 [file ijms-25-11843-s001.zip › Table S25_Samples_2024.10.26.pdf]

Supplementary Table S25. Clinical and histological characteristics for 140 primary ovarian tumors examined in the study.

#	Stage	TNM	Histological type	Grade	Tumors with PM	Ten lncRNA level	Four miRNA level	Seven mRNA level	Alive/Died patients
1	I	T1N0M0	Serous adenocarcinoma	1					++
2	I	T1N0M0	Serous adenocarcinoma	1					++
3	III	T3cN1M0	Serous adenocarcinoma	3					+
4	III	T3N1M0	Serous adenocarcinoma	3		+			+
5	III	T3N0M0	Endometrioid adenocarcinoma	3					+
6	II	T2cN0M0	Serous adenocarcinoma	2		+			++
7	IV	T3cN1M1	Serous adenocarcinoma	3					+
8	I	T1cN0M0	Serous adenocarcinoma	1					+
9	I	T1cN0M0	Serous adenocarcinoma	1					+
10	IIIc	T3cN0M0	Serous adenocarcinoma	3		+			+
11	IIIc	T3N1M0	Serous adenocarcinoma	3		+			+
12	III	T3N0M0	Serous adenocarcinoma	3		+			+
13	IIIc	T3N1M0	Serous adenocarcinoma	2		+			+
14	I	T1cN0M0	Endometrioid adenocarcinoma	1					+
15	III	T3cN0M0	Serous adenocarcinoma	3					++
16	III	T3aN0M0	Serous adenocarcinoma	2		+	+		++
17	III	T3N0M0	Serous adenocarcinoma	1		+			++
18	III	T3N0M0	Serous adenocarcinoma	1					++
19	IV	T3cN1M1	Serous adenocarcinoma	3		+	+		++
20	I	T1cN0M0	Endometrioid adenocarcinoma	2		+			++
21	II	T2cN0M0	Serous adenocarcinoma	1		+	+		++
22	IIIc	T3cN0M0	Serous adenocarcinoma	2		+			++
23	I	T1aN0M0	Endometrioid adenocarcinoma	2		+			++
24	II	T2cN0M0	Clear cell adenocarcinoma	3					++
25	I	T1N0M0	Endometrioid adenocarcinoma	2		+			++
26	I	T1N0M0	Serous adenocarcinoma	3					++
27	IIIc	T3cN0M0	Serous adenocarcinoma	3		+			++
28	II	T2cN0M0	Serous adenocarcinoma	1					++
29	II	T2cN0M0	Serous adenocarcinoma	3					++
30	I	T1N0M0	Serous adenocarcinoma	1		+	+		++
31	IIIc	T3cN0M0	Serous adenocarcinoma	2					++
32	IIIc	T3cN0M0	Serous adenocarcinoma	3		+			+
33	IIIc	T3cN1M0	Serous adenocarcinoma	3		+			++
34	IIIc	T3cN0M0	Serous adenocarcinoma	2		+			+
35	IIIc	T3cN1M0	Serous adenocarcinoma	3		+			+
36	IIIc	T3cN1M0	Serous adenocarcinoma	3					+
37	II	T2cN0M0	Serous adenocarcinoma	1					+
38	IIIc	T3cN1M0	Serous adenocarcinoma	3					+
39	III	T3N0M0	Endometrioid adenocarcinoma	2		+	+		++
40	III	T3M0N0	Serous adenocarcinoma	3		+			++
41	III	T3bN0M0	Serous adenocarcinoma	1					++
42	II	T2N0M0	Serous adenocarcinoma	3					++
43	IIIc	T3cN0M0	Serous adenocarcinoma	2					+
44	II	T2cN0M0	Serous adenocarcinoma	3	+	+			++
45	II	T2N0M0	Endometrioid adenocarcinoma	2		+	+		++

46	III	T1N1M0	Endometrioid adenocarcinoma	2		+	+	+	++
47	IIIc	T3N1M0	Serous adenocarcinoma	1		+	+	+	++
48	III	T3cN1M0	Serous adenocarcinoma	3		+			++
49	II	T2N1M0	Serous adenocarcinoma	2	+	+		++	++
50	IV	T3cN0M1	Serous adenocarcinoma	1	+	+	+	++	++
51	IV	T3cN0M1	Mucinous adenocarcinoma	3	+	+		++	++
52	IIIc	T3cN0M0	Serous adenocarcinoma	1		+	+	+	++
53	IV	T3cN1M1	Endometrioid adenocarcinoma	3	+	+	+	++	++
54	III	T3bN0M0	Serous adenocarcinoma	1		+		+	++
55	IV	T3cN0M1	Serous adenocarcinoma	1	+	+	+	++	++
56	IV	T3bN0M1	Clear cell adenocarcinoma	3	+	+	+	++	++
57	IV	T1N0M1	Mucinous adenocarcinoma	4	+	+		++	++
58	IIIc	T2bN1M0	Serous adenocarcinoma	1	+	+	+	++	++
59	IIIc	T2bN1M0	Serous adenocarcinoma	1	+	+		+	++
60	IIIc	T3bN1M0	Serous adenocarcinoma	1	+	+	+	++	++
61	IV	T3cN1M1	Serous adenocarcinoma	1	+	+	+	++	++
62	III	T3aN0M0	Serous adenocarcinoma	1	+	+		++	++
63	IIIc	T3cN1M0	Endometrioid adenocarcinoma	3	+	+	+	++	++
64	I	T1bN0M0	Serous adenocarcinoma	1		+	+	+	++
65	IIIc	T3cN1M0	Serous adenocarcinoma	3		+		+	++
66	IV	T3cN1M1	Serous adenocarcinoma	1	+	+	+	++	++
67	IIIc	T3cN0M0	Serous adenocarcinoma	3	+	+	+	++	++
68	I	T1aN0M0	Mucinous adenocarcinoma	1		+	+	+	++
69	III	T3bN1M0	Serous adenocarcinoma	1	+	+	+	++	++
70	IV	T3cN0M1	Serous adenocarcinoma	3	+	+		++	++
71	IIIc	T3cN0M0	Serous adenocarcinoma	1	+	+	+	++	++
72	IV	T3cN0M1	Serous adenocarcinoma	3	+	+	+		++
73	IIIc	T3cN0M0	Clear cell adenocarcinoma	3		+	+	+	+
74	IV	T3cN0M1	Undifferentiated carcinoma	4					++
75	IIIc	T3cN0M0	Serous adenocarcinoma	2	+				+
76	IIIc	T3cN0M0	Serous adenocarcinoma	2	+				+
77	II	T2bN0M0	Serous adenocarcinoma	2	+				+
78	IIIc	T3cN0M0	Serous adenocarcinoma	3	+				++
79	IIIc	T3cN0M0	Serous adenocarcinoma	3	+				++
80	IIIc	T3cN0M0	Serous adenocarcinoma	3	+				++
81	IIIc	T3cN0M0	Serous adenocarcinoma	3	+				++
82	I	T1cN0M0	Serous adenocarcinoma	2	+				++
83	I	T1cN0M0	Serous adenocarcinoma	2	+	+			+
84	I	T1cN0M0	Serous adenocarcinoma	2	+	+			++
85	III	T3aN0M0	Serous adenocarcinoma	2					++
86	II	T2cN0M0	Endometrioid adenocarcinoma	2					+
87	I	T1cN0M0	Serous adenocarcinoma	2		+			+
88	III	T3bN0M0	Serous adenocarcinoma	3		+			+
89	III	T3aN0M0	Serous adenocarcinoma	2		+			++
90	IIIc	T3cN0M0	Serous adenocarcinoma	3		+			++
91	IIIc	T3cN0M0	Serous adenocarcinoma	2		+			+
92	IIIc	T3cN0M0	Serous adenocarcinoma	3	+				++
93	I	T1cN0M0	Endometrioid adenocarcinoma	1		+			++
94	IIIc	T3cN0M0	Serous adenocarcinoma	3	+				++

95	III	T3aN1M0	Serous adenocarcinoma	1		+			++
96	IIIc	T3cN0M0	Serous adenocarcinoma	2	+				++
97	I	T1cN0M0	Serous adenocarcinoma	1		+			++
98	I	T1aN0M0	Serous adenocarcinoma	2		+			++
99	I	T1cN0M0	Endometrioid adenocarcinoma	2		+			++
100	II	T2bN0M0	Endometrioid adenocarcinoma	3		+			+
101	IIIc	T3cN0M0	Serous adenocarcinoma	3		+			++
102	II	T2bN0M0	Endometrioid adenocarcinoma	2		+			++
103	II	T2cN0M0	Serous adenocarcinoma	3		+			++
104	I	T1cN0M0	Mucinous adenocarcinoma	2		+			++
105	IIIc	T3cN0M0	Serous adenocarcinoma	3	+				++
106	IIIc	T3cN0M0	Serous adenocarcinoma	3	+				++
107	IIIc	T3cN0M0	Serous adenocarcinoma	3	+				++
108	IIIc	T3cN0M0	Serous adenocarcinoma	3	+				++
109	IIIc	T3cN0M0	Serous adenocarcinoma	3	+				+
110	IIIc	T3cN0M0	Serous adenocarcinoma	3	+				++
111	IIIc	T3cN0M0	Endometrioid adenocarcinoma	3	+				+
112	IIIb	T3bN0M0	Serous adenocarcinoma	1	+				++
113	II	T2bN0M0	Serous adenocarcinoma	1		+	+		++
114	I	T1cN0M0	Mucinous adenocarcinoma	1		+	+		++
115	I	T1cN0M0	Endometrioid adenocarcinoma	2		+	+		++
116	I	T1cN0M0	Clear cell adenocarcinoma	3		+			+
117	III	T3bN0M0	Serous adenocarcinoma	3	+				++
118	IIIc	T3cN0M0	Mucinous adenocarcinoma	3	+				+
119	II	T2cN0M0	Endometrioid adenocarcinoma	2	+				+
120	I	T1cN0M0	Mucinous adenocarcinoma	1		+			++
121	III	T3bN0M0	Serous adenocarcinoma	2	+				+
122	II	T2cN0M0	Serous adenocarcinoma	2	+				+
123	II	T2bN0M0	Endometrioid adenocarcinoma	3	+				+
124	III	T3bN0M0	Serous adenocarcinoma	3	+				++
125	IIIc	T3cN0M0	Serous adenocarcinoma	3	+	+	+	+	++
126	II	T2cN0M0	Mucinous adenocarcinoma	2		+	+		+
127	IV	T3cN1M1	Serous adenocarcinoma	3	+	+	+		++
128	IIIc	T3cN1M0	Serous adenocarcinoma	3	+	+	+		++
129	IV	T3cN1M1	Endometrioid adenocarcinoma	3	+	+	+		++
130	IIIc	T3cN0M0	Serous adenocarcinoma	1	+	+	+		++
131	IIIc	T3cN0M0	Serous adenocarcinoma	3	+	+	+		++
132	IIIc	T3cN0M0	Serous adenocarcinoma	3		+	+		++
133	IIIc	T3cN1M0	Serous adenocarcinoma	3		+			++
134	II	T2bN0M0	Serous adenocarcinoma	1	+	+	+		++
135	IIIc	T3cN0M0	Endometrioid adenocarcinoma	3	+	+	+		++
136	IIIc	T3cN1M0	Serous adenocarcinoma	3	+	+			++
137	IIIc	T3cN0M0	Serous adenocarcinoma	3		+	+		++
138	II	T2bN0M0	Serous adenocarcinoma	3		+	+		++
139	IIIc	T3cN1M0	Serous adenocarcinoma	2	+	+	+		++
140	IIIc	T3cN0M0	Serous adenocarcinoma	3	+	+			++

Note: nd – no data; 59 primary tumor samples studied with peritoneal macroscopic metastases (PM) are marked by "+" and pale pink shading; 90 primary tumor samples examined in expression studies of 10 lncRNAs are marked by "+" and pale green shading; 41 primary tumor samples examined in expression studies of 4 miRNAs (miR-124-3p, miR-124-5p, miR-137-3p, miR-33b-5p) are marked by "+" and pale gray shading; 27 primary tumor (+) and 17 PM (++)

samples examined in expression studies of 7 mRNAs are marked by yellow shading; 37 patients who died are marked by “+” and pale blue shading and 103 patients who are alive are marked by “++” and dark blue shading.
