# Supplementary material for: Ten Hypermethylated lncRNA Genes Are Specifically Involved in the Initiation, Progression, and Lymphatic and Peritoneal Metastasis of Epithelial Ovarian Cancer
Source: Int J Mol Sci. 2024 Nov 4;25(21):11843. doi: 10.3390/ijms252111843 (PMC11547154; doi:10.3390/ijms252111843)
Supplement: Supplementary file 1 [file ijms-25-11843-s001.zip › Table S26_Primers_qPCR_2024.10.26.pdf]

Supplementary Table S26. Primers and PCR conditions in the RT-qPCR study.

Gene	Primer sequence (5'-3')	Tann, °C	Product size, bp
B2M	F: TGACTTTGTCACAGCCCAAGATAG	60	81
	R: CAAATGCGGCATCTTCAAACCTC		
**LncRNAs**			
GAS5	F: GAGCAAGCCTAACTCAAGCC	59	157
	R: TCAAGCCGACTCTCCATACCC		
HAND2-AS1	F: CCCCGAATCTGTAGTGTGGC	59	113
	R: CAGGCGGTGGAGAGGACT		
KCNK15-AS1	F: GGGCGGCTCACCGATGGTAGT	59	237
	R: CTGGTGGGCGCTGCTGTCTTC		
MAGI2-AS3	F: CGCTGACTCCCTCCGACCTG	59	155
	R: GCATCCTTTTGCCCCTGTCC		
MEG3	F: CGGCTGGGTCGGCTGAAGAACT	59	208
	R: CCGTGGCTGTGGAGGGATTT		
SEMA3B-AS1	F: AGCAGGCAAAGGGAATCACTGAGT	59	184
	R: ACCCCAGGTCATTCCCAACTGA		
SNHG6	F: GTAGCTGGGCTCTGCGAGGTG	59	213
	R: GCTGCATGCCACACTTGAGGTAAC		
SSTR5-AS1	F: ACTTTGGGATGTGGCTGGATGTC	59	144
	R: ATGCCCCTAAATGTGGAAATGTGAAG		
ZEB1-AS1	F: GAACCGGGATGGGAAGTGACT	59	144
	R: GTGCGGATGGGGAAGTGAGA		
ZNF667-AS1	F: ACCGTTGCGTAATTGTGAGTCTGTGA	59	219
	R: CTTATCCGGGAATGAGTTGGTTGTTG		
**mRNAs**			
FKBP14	F: GCC ATC GCA AGA CCA AAG GAG	59	223
	R: CCA TAG CCC AGA GCA GGA GGA A		
SERPINF1-iso1	F: CGC TGG TGG AGG AGG AGG AT	59	212
	R: GAG AGC CCG GTG AAT GAT GGA		
SNAI2/SLUG	F: AGA TGC CGC GCT CCT TCC TG	59	173
	R: AGT GAT GGG GCT GTA TGC TCC TGA		
CDH1	F: ACG GGA ATG CAG TTG AGG A	59	186
	R: TGG CGG CAT TGT AGG TGT T		
VIM	F: CGC CAG GCA AAG CAG GAG TC	59	170
	R: TGC AGG CGG CCA ATA GTG TCT		
ZEB1	F: CAG GCA GAT GAA GCA GGA TG	59	243
	R: CAG CAG TGT CTT GTT GTT GTA G		
ZEB2	F: AGC AGG TAA TCG CAA GTT CAA	59	116
	R: AGT TTG GGC ACT CGT AAG GTT		

Note: F – forward primer; R – reverse primer.
