# Supplementary material for: Ten Hypermethylated lncRNA Genes Are Specifically Involved in the Initiation, Progression, and Lymphatic and Peritoneal Metastasis of Epithelial Ovarian Cancer
Source: Int J Mol Sci. 2024 Nov 4;25(21):11843. doi: 10.3390/ijms252111843 (PMC11547154; doi:10.3390/ijms252111843)
Supplement: Supplementary file 1 [file ijms-25-11843-s001.zip › Table S27_Primers_MS-qPCR_2024.10.26.pdf]

Supplementary Table S27. Primers and PCR conditions in the quantitative methylation-specific PCR (MS-qPCR) study.

lncRNA gene	M/U	Primer sequence (5'-3')	Product length, bp	Tann., °C
GAS5	M	F: CGTTATCGTCGGTATTGGAGGGG	60	185
		R: CGCCCGACGCCTTATCCC		
	U	F: TGTTATTGTTGGTATTGGAGGGGTGAG	60	179
		R: CAACACCTTATCCCCATCTTCTCCA		
HAND2-AS1	M	F: CGAGGTTGGTACGCGGAG	60	121
		R: CCGACACAACTAAACCGACTC		
	U	F: TGGGGTTTTTGTGAGGTTGGTATGT	60	134
		R: CCCCAACACAACTAAACCAACTCCTC		
KCNK15-AS1	M	F: CGGTGATGGCGAAGTAGAAGGAGT	60	182
		R: CGAATCCGAAACGAAAAACGACC		
	U	F: GATGATGGTGATGGTGAAGTAGAAGGAGT	60	163
		R: CCAACAACTACTAATCCAAAAACAAAACACTC		
MAGI2-AS3	M	F: CGGAGCGAGTAGTAGTCGAGTTGGT	60	276
		R: CGACGAAACCCTCCGTAACTCC		
	U	F: TGGAGTGAGTAGTAGTTGAGTTGGTGAGTG	59.4	330
		R: CTCTCTCTCTACCTTCACTACCAAATCAAACTAC		
MEG3	M	F: CGTTAAGTTCGTATTTTTCGATGGATGTT	60	185
		R: CGCGAATACTTTTTCCCTACGTAAACC		
	U	F: TGATGGATGTTTTGAAATTGTTAGGTGTG	60	165
		R: CAAATACTTTTTCCCTACATAAACCCAACTCA		
SEMA3B-AS1	M	F: CCACTCCCGCCTAACTACCG	54	91
		R: ATCGTTCGTCGTGTCGTAAAGT		
	U	F: ACTCCCACCTAACTACCA	46	90
		R: TATTGTTTGTTGTGTTGTAA		
SNHG6	M	F: TTGAGTTATCGCGTTCGGTTT	61	295
		R: CTCTTCCGATACGCGACCC		
	U	F: TTGAGTTATTGTGTTTGGTTT	58	295
		R: CTCTTCCAATACACAACCC		
SSTR5-AS1	M	F: CGGCGTTAGCGGGTCGAGT	59.4	153
		R: CGCTCCTTCTAACCCTTCGAC		
	U	F: TGTGGGTGGTGTTAGTGGGTTGAGT	60	168
		R: ACAAAACACCACATCCTTCTAACCCTTC		
ZEB1-AS1	M	F: CGGTCGGCGGTAGGGTCG	60	120
		R: CGAAAAAAACGAAACTTATCCGCC		
	U	F: TGTAGGAATTAAAGTGAGTTTTTTGGTTTGAA	60	185
		R: CAAAAAAAACAAAACTTATCCACCCAAA		
ZNF667-AS1	M	F: AGGCGCGAGTTTATCGTTTAC	60	254
		R: ACGCGCGATCCCGAAAT		
	U	F: AGGTGTGAGTTTATTGTTTATGTA	58	260
		R: AACACACAATCCCAAAATCCC		

Note: M – methylated allele; U – unmethylated allele; F – forward primer; R – reverse primer.
